# Flavodoxin-Like Proteins Protect *Candida albicans* from Oxidative Stress and Promote Virulence

**DOI:** 10.1371/journal.ppat.1005147

**Published:** 2015-09-01

**Authors:** Lifang Li, Shamoon Naseem, Sahil Sharma, James B. Konopka

**Affiliations:** Department of Molecular Genetics and Microbiology, Stony Brook University, Stony Brook, New York, United States of America; University of Würzburg, GERMANY

## Abstract

The fungal pathogen *Candida albicans* causes lethal systemic infections in humans. To better define how pathogens resist oxidative attack by the immune system, we examined a family of four Flavodoxin-Like Proteins (FLPs) in *C*. *albicans*. In agreement with previous studies showing that FLPs in bacteria and plants act as NAD(P)H quinone oxidoreductases, a *C*. *albicans* quadruple mutant lacking all four FLPs (*pst1Δ*, *pst2Δ*, *pst3Δ*, *ycp4Δ*) was more sensitive to benzoquinone. Interestingly, the quadruple mutant was also more sensitive to a variety of oxidants. Quinone reductase activity confers important antioxidant effects because resistance to oxidation was restored in the quadruple mutant by expressing either *Escherichia coli wrbA* or mammalian *NQO1*, two distinct types of quinone reductases. FLPs were detected at the plasma membrane in *C*. *albicans*, and the quadruple mutant was more sensitive to linolenic acid, a polyunsaturated fatty acid that can auto-oxidize and promote lipid peroxidation. These observations suggested that FLPs reduce ubiquinone (coenzyme Q), enabling it to serve as an antioxidant in the membrane. In support of this, a *C*. *albicans coq3Δ* mutant that fails to synthesize ubiquinone was also highly sensitive to oxidative stress. FLPs are critical for survival in the host, as the quadruple mutant was avirulent in a mouse model of systemic candidiasis under conditions where infection with wild type *C*. *albicans* was lethal. The quadruple mutant cells initially grew well in kidneys, the major site of *C*. *albicans* growth in mice, but then declined after the influx of neutrophils and by day 4 post-infection 33% of the mice cleared the infection. Thus, FLPs and ubiquinone are important new antioxidant mechanisms that are critical for fungal virulence. The potential of FLPs as novel targets for antifungal therapy is further underscored by their absence in mammalian cells.

## Author Summary

Oxidative damage is a fundamental problem for cells and a particular challenge for microbial pathogens, which require special mechanisms to resist the oxidative attack by the host immune system. We identified four proteins in the human fungal pathogen *Candida albicans* that belong to a large family of enzymes in bacteria and plants that reduce quinone molecules to detoxify them. Interestingly, mutational studies in *C*. *albicans* showed that these enzymes also confer resistance to a wide range of oxidants, suggesting they may have broader impact by reducing the major quinone present in cells (ubiquinone or coenzyme Q). In support of this, we found that mutating the *COQ3* gene to block ubiquinone synthesis rendered cells highly sensitive to oxidative stress, revealing that it plays a very important antioxidant function in addition to its well known role in energy metabolism. These quinone reductases play a critical role in vivo, as they were required for virulence in mouse infections studies. Since mammalian cells lack this type of quinone reductase, this difference could be exploited to develop much needed novel therapeutic approaches for fungal and bacterial pathogens.

## Introduction

Oxidative stress poses a great threat to cells. Unchecked oxidative damage to DNA, proteins, and lipids causes disruption of physiological processes, harmful mutations, and cell death [1]. To prevent these destructive effects, cells utilize a variety of mechanisms to protect against oxidation. These antioxidant mechanisms are especially important for pathogens to resist the oxidative attack by the immune system [2]. As a result, the human fungal pathogen *Candida albicans* relies on several different mechanisms, such as extracellular, cytoplasmic, and mitochondrial forms of superoxide dismutases to break down superoxide radicals [3–5]. Other intracellular mechanisms include catalase to detoxify H_2_O_2_ and glutathione to promote a reducing environment [6].

Cellular membranes require special protection from oxidation. The plasma membrane is particularly vulnerable because it directly faces oxidative attack by macrophages and neutrophils. Protecting the plasma membrane is critical for survival. In addition to forming a protective barrier around the cell, it functions in a wide range of essential processes including nutrient uptake, ion homeostasis, pH regulation, cell wall synthesis, and morphogenesis. This membrane is also vulnerable because it contains polyunsaturated fatty acids (PUFAs). Approximately 30% of the *C*. *albicans* fatty acids are polyunsaturated linoleic (18:2) or linolenic (18:3) acids [7, 8]. PUFAs are very sensitive to peroxidation due to the ease with which the hydrogens can be abstracted from the methylene bridges (-CH_2_-) that lie in between the double bonds [9, 10]. This leaves an unpaired electron on the carbon that can react with O_2_ to form a peroxyl radical, which can in turn abstract the hydrogen from another PUFA to continue the cycle. Thus, lipid peroxidation starts a chain reaction that propagates to other lipids. The resulting oxidative damage can also spread to other cellular constituents, including proteins and DNA.

Several lines of evidence suggested that a family of four uncharacterized Flavodoxin-Like Proteins (FLPs) present in *C*. *albicans* could play a novel antioxidant role at the plasma membrane. The FLPs, which are encoded by *PST1*, *PST2*, *PST3*, and *YCP4*, are induced by oxidative stress [11]. The FLP genes contain consensus sites in their promoter regions for the binding of Cap1, a transcription factor that is induced by oxidative stress, and for a subset of these genes Cap1 has been shown to bind to the promoter and regulate expression [12, 13]. The *S*. *cerevisiae* FLPs (Pst2, Rfs1, Ycp4) have been suggested to promote resistance to oxidative stress [14–16], although their physiological role is not known [17]. It is also interesting that the *C*. *albicans* FLPs are likely to act at the plasma membrane, since their orthologs in *S*. *cerevisiae* are associated with the plasma membrane [18].

**Fig 1:**
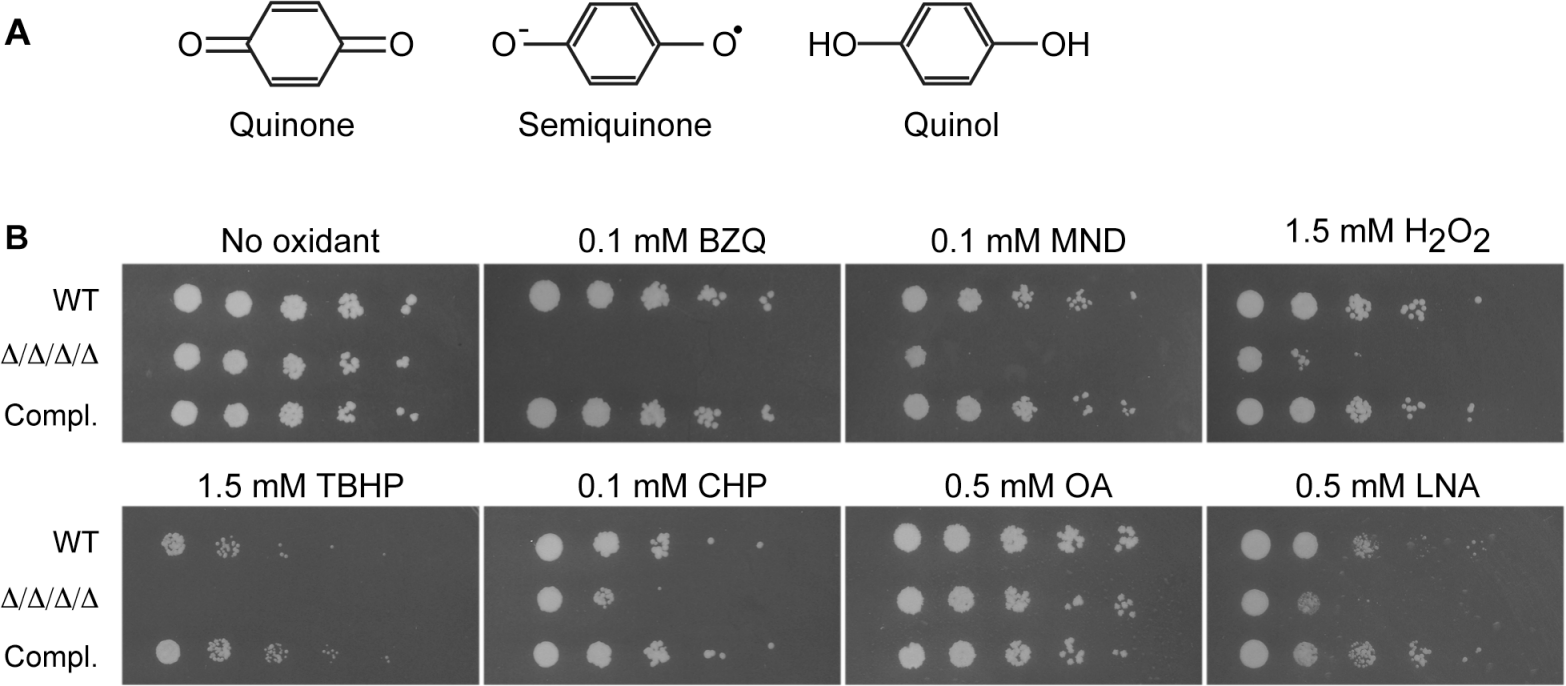
*C*. *albicans* FLP genes are needed for resistance to oxidative stress. (A) Structures of oxidized benzoquinone, reduced benzoquinol, and the semiquinone intermediate that is a reactive oxygen species. Two-electron reduction of quinones avoids creation of the semiquinone intermediate. (B) Serial 10-fold dilutions of cells were spotted onto synthetic medium agar plates containing the indicated chemical and then incubated at 37°C for 48 h. Quinones tested included BZQ (p-benzoquinone) and MND (menadione). Oxidants included H_2_O_2_, as well as more hydrophobic peroxides that target the membrane: TBHP (tert-butyl hydroperoxide) and CHP (cumene hydroperoxide). In addition, monounsaturated OA (oleic acid) was used as a control for the polyunsaturated LNA (linolenic acid), which is known to induce lipid peroxidation. Strains used were the wild type strain LLF100, Δ/Δ/Δ/Δ strain LLF060 (*pst1Δ pst2Δ pst3Δ ycp4Δ*), and the complemented strain LLF079 in which one copy of each FLP gene was introduced into the Δ/Δ/Δ/Δ strain (*pst1Δ pst2Δ pst3Δ ycp4Δ PST1 PST2 PST3 YCP4*).

The FLPs are highly conserved in bacteria, plants, and fungi, but surprisingly not in mammalian cells [19]. Biochemically, the most well studied FLP is the *E*. *coli* WrbA protein. It uses flavin mononucleotide (FMN) as a cofactor and acts as a NAD(P)H quinone oxidoreductase [20–22]. FLPs from fungi, plants and other bacteria have also been shown to act as NAD(P)H quinone oxidoreductases, indicating that this is a conserved property of this family [15, 23–27]. A special feature of FLPs is that they carry out a two-electron reduction of a quinone to quinol (see structures in Fig 1A). This converts both carbonyl groups on the benzoquinone ring to hydroxyl groups. In contrast, other pathways that promote a one-electron reduction of quinone form a semiquinone intermediate that is a hazardous reactive oxygen species [9, 10]. Although the physiological role of WrbA is not known, there is suggestive evidence that it promotes resistance to oxidative stress [19, 21, 27].

Quinone reductases could promote resistance to oxidative stress in several ways. One is that they can reduce and detoxify small molecule quinones that are produced by some organisms for defense or created as benzene metabolites [28, 29]. In addition, they could act on endogenously produced quinones, such as ubiquinone (coenzyme Q), an isoprenylated benzoquinone. Ubiquinone is well known for its role in the mitochondrial electron transport chain, but it is also present in other cellular membranes, where it can undergo redox cycling to act as an antioxidant [30–34]. Mammalian cells use the enzyme Nqo1 (NAD(P)H quinone oxidoreductase), formerly known as DT-diaphorase, to safely carry out a two-electron reduction of ubiquinone and avoid semiquinone formation [35, 36]. Nqo1 is analogous to FLPs in that it uses NAD(P)H for reducing potential, but it differs in overall amino acid sequence and the active site is distinct from the FLPs, in part due to the fact that the active site of Nqo1 binds FAD as a cofactor rather than FMN [19]. However, it is not known how fungal cells, including *C*. *albicans*, carry out this function since they lack an obvious ortholog of *NQO1*. Therefore, in this study we examined a quadruple mutant lacking all four FLP genes (*PST1*, *PST2*, *PST3* and *YCP4*). The results demonstrate that these proteins represent a new mechanism for protecting *C*. *albicans* against oxidative stress that is required for virulence in a mouse model of systemic candidiasis.

## Results

### *C*. *albicans* mutant lacking all four FLP genes is more sensitive to oxidation

Four FLPs were identified in *C*. *albicans* based on their high sequence identity (45–50%) and similarity (~65%) to the well-studied *E*. *coli* WrbA (S1 Fig). This type of enzyme is advantageous because it uses NAD(P)H to carry out a two-electron reduction of toxic quinones that avoids creation of the semiquinone radical (Fig 1A) [21]. The conserved residues are concentrated in the active site near the location of the FMN co-factor. The four *C*. *albicans* FLPs share a similar structure, although Ycp4 contains C-terminal extension of about 90 amino acids that ends in a CAAX box, indicating it is likely to be lipid modified (S1 Fig). To examine their role in the diploid *C*. *albicans*, a quadruple mutant strain was constructed that lacks both copies of all four FLP genes. Fortuitously, *PST3* and *YCP4* are adjacent in the genome and were deleted simultaneously using the *HIS1* and *LEU2* selectable markers. Subsequent deletion of the *PST1* and *PST2* genes was carried out by successive use of the SAT Flipper that employs a recyclable *SAT1* selectable marker [37]. For brevity, this *pst1Δ pst2Δ pst3Δ ycp4Δ* strain will be referred to as the Δ/Δ/Δ/Δ mutant. The sensitivity of this strain to quinones was tested by spotting dilutions of cells onto agar medium containing p-benzoquinone (BZQ) or menadione (MND), a heterocyclic napthoquinone (Fig 1B). The growth of the Δ/Δ/Δ/Δ strain was clearly inhibited by these small molecule quinones, indicating it is more sensitive to quinones than either the wild type control or a complemented strain in which one copy of each of the FLP genes was reintroduced.

FLPs in bacteria and plants have also been suggested to have a role in fighting oxidative stress, but their physiological role is not known [19, 21, 25, 27]. Therefore, given the importance of antioxidant enzymes for microbial pathogens, we spotted the cells on medium containing H_2_O_2_ and found that the Δ/Δ/Δ/Δ mutant was more sensitive to this oxidant (Fig 1B). Since the FLPs are associated with the plasma membrane in *S*. *cerevisiae* [18], we further tested two other peroxides that are more hydrophobic. Interestingly, the Δ/Δ/Δ/Δ mutant was also very sensitive to tert-butyl hydroperoxide (TBHP) and cumene hydroperoxide (CHP), which are more hydrophobic than H_2_O_2_ and more likely to preferentially oxidize membranes.

The Δ/Δ/Δ/Δ mutant was next assayed for sensitivity to polyunsaturated fatty acids (PUFAs), which can auto-oxidize and initiate a chain reaction of lipid peroxidation [10, 33]. PUFAs are more readily oxidized because the presence of double bonds flanking a methylene group (-CH_2_-) weakens the methylene C-H bond, making it much easier to abstract a hydrogen [9]. This leaves a carbon with an unpaired electron that readily reacts with oxygen to form a peroxyl radical (LOO•). For example, linolenic acid, which has three unsaturated double bonds, is much more likely to auto-oxidize to form a peroxyl radical than is monounsaturated oleic acid. The peroxyl radical can then abstract a hydrogen from another PUFA to form a lipid peroxide (LOOH) and a new lipid radical that can further extend a chain reaction of lipid peroxidation [9, 10]. Linolenic acid was also used for this analysis because previous studies showed that it efficiently induced lipid peroxidation and cell death in *S*. *cerevisiae* [33]. Interestingly, growth of the Δ/Δ/Δ/Δ mutant was strongly inhibited by the polyunsaturated linolenic acid (LNA; Fig 1B). In contrast, the Δ/Δ/Δ/Δ mutant grew as well as the control cells in the presence of the monounsaturated oleic acid (OA). Taken together, these results indicate that the FLPs are needed for *C*. *albicans* to combat a variety of oxidative stresses.

### FLPs are needed to prevent lipid peroxidation

**Fig 2:**
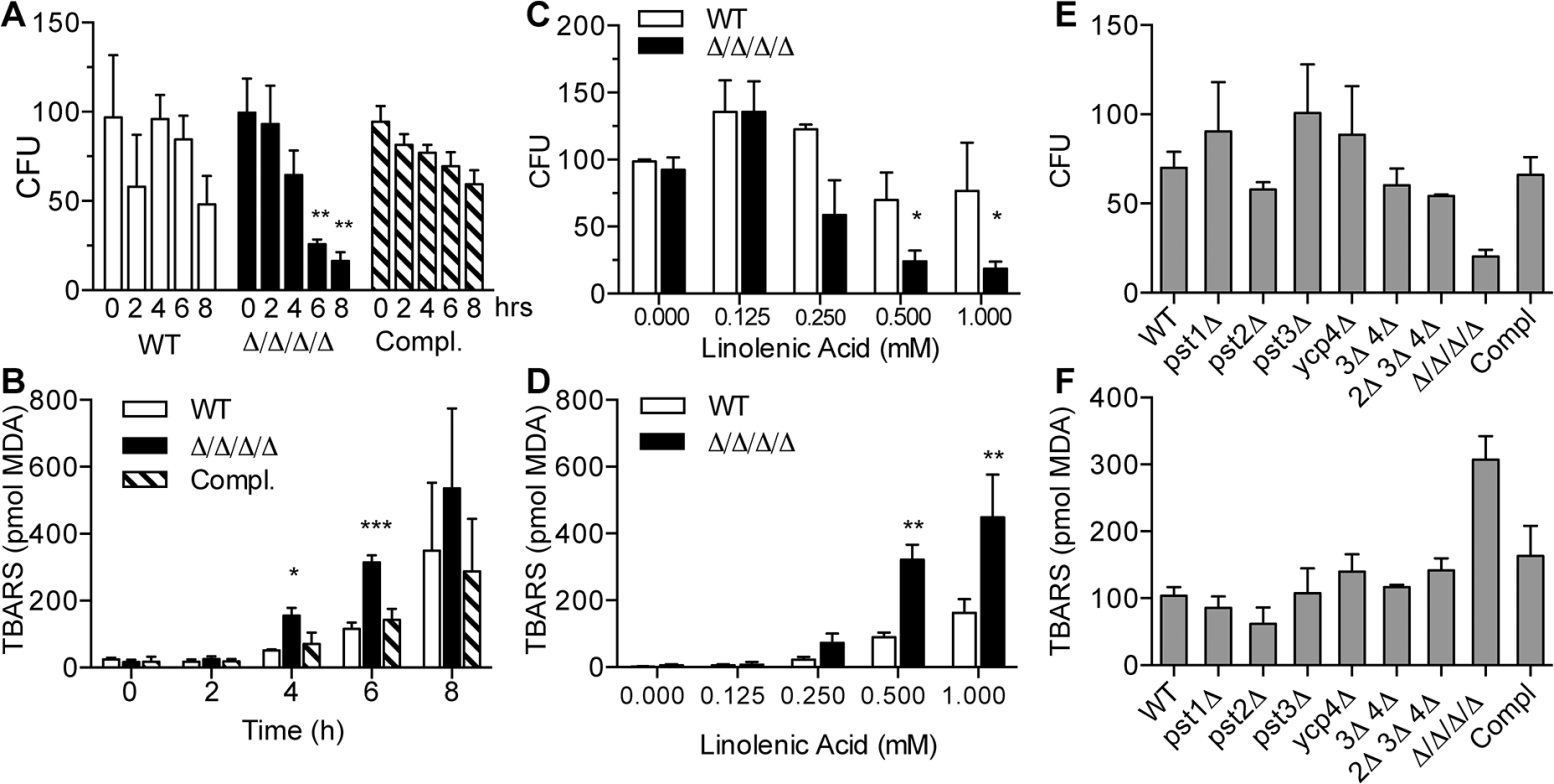
The Δ/Δ/Δ/Δ mutant strain is more sensitive to linolenic acid-induced cell death and lipid peroxidation. (A) *C*. *albicans* strains were incubated with 0.5 mM linolenic acid (LNA) at 37°C for the indicated time (hours), and then dilutions of cells were plated to determine the viable colony forming units (CFU). (B) Cells were incubated with 0.5 LNA for the different times and then thiobarbituric acid reactive substance (TBARS) assays were carried out to detect malondialdehyde (MDA), a byproduct of lipid peroxidation. (C) Cells were exposed to different concentrations of LNA for 6 h, and then CFUs were determined. (D) TBARS assays to detect lipid peroxidation in cells treated with different concentrations of LNA for 6 h. (E) CFU analysis and (F) TBARS assays of the indicated FLP mutant strains. Note that in contrast to the Δ/Δ/Δ/Δ quadruple mutant strain LLF060, the single, double and triple FLP deletion mutant strains did not display increased sensitivity to LNA. Error bars indicate SE. * = p<0.05, ** = p<0.01, *** = p< 0.001 by ANOVA. Strains used included the wild type strain LLF100, Δ/Δ/Δ/Δ strain LLF060, and the complemented strain LLF079 in which one copy of each FLP gene was introduced into the Δ/Δ/Δ/Δ strain.

The effects of linolenic acid on *C*. *albicans* were analyzed further in quantitative assays. A time course of cell death was assayed by incubating cells for different times in the presence of 0.5 mM linolenic acid followed by plating dilutions on agar medium to determine the viable colony forming units (CFUs). The results confirmed the spotting assays. The Δ/Δ/Δ/Δ mutant showed a significant trend toward decreased viability by 6–8 h that was not observed for the wild-type control or complemented strains (Fig 2A). Analysis of the dose-response to incubation with linolenic acid for 6 h revealed a loss of viability starting at 0.25 mM that became more significant at 0.5 and 1.0 mM (Fig 2C). In contrast, the cells remained viable after incubation in the monounsaturated oleic acid (S2 Fig).

To determine whether linolenic acid caused an increase in lipid peroxidation, cells were assayed for thiobarbituric acid reactive substances (TBARS) [33, 38]. This assay detects malondialdehyde (MDA), a common byproduct of lipid peroxidation. As expected, both the Δ/Δ/Δ/Δ mutant and the control cells showed elevated TBARS after incubation for different times with linolenic acid (Fig 2B). However, the Δ/Δ/Δ/Δ mutant showed a significantly higher level of TBARS than the control cells at 4 and 6 h. By 8 h, the results of the TBARS assays were quite variable. This may have been due to difficulties in dealing with the high fraction of dead cells during the analysis. Dose-response assays showed that the TBARS in the Δ/Δ/Δ/Δ mutant started trending upward at 0.25 mM and was significantly higher than control cells at 0.5 mM and 1.0 mM linolenic acid (Fig 2D). These results demonstrate that linolenic acid stimulated higher levels of lipid peroxidation in the Δ/Δ/Δ/Δ mutant.

For comparison, mutants lacking a single FLP gene (*pst1Δ*, *pst2Δ*, *pst3Δ or ycp4Δ*), two genes (*pst3Δ ycp4Δ*), or three genes (*pst2Δ*, *pst3Δ ycp4Δ*) were also tested for their sensitivity to 0.5 mM linolenic acid (Fig 2E and 2F). However, no significant changes in either CFU or lipid peroxidation level were detected compared to the wild type control. As will be described further below, this is consistent with redundancy of the different FLP genes in *C*. *albicans*.

**Fig 3:**
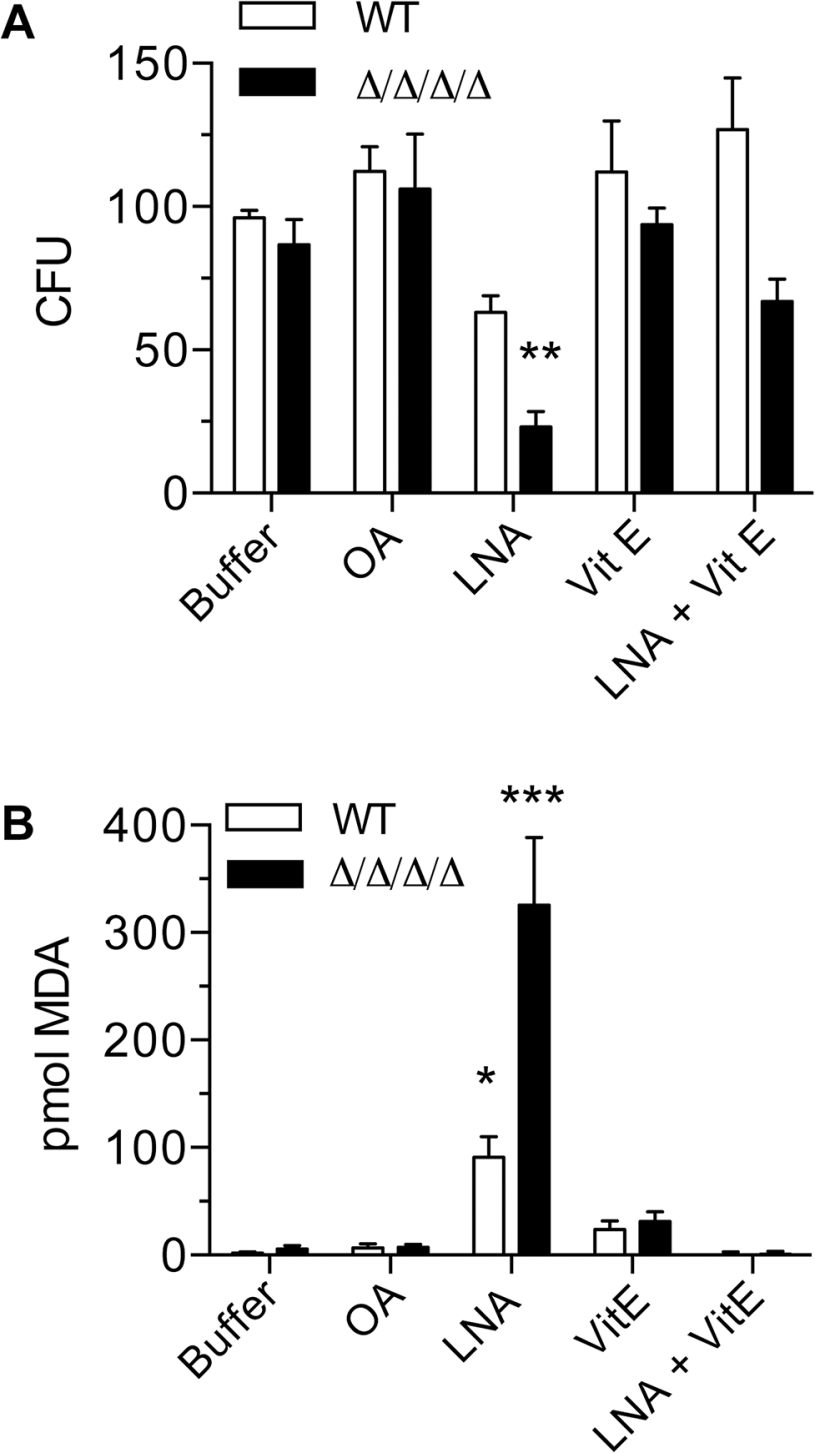
Antioxidant Vitamin E blocks the effects of linolenic acid. Cells were suspended in phosphate buffer (0.1M Na_2_HPO_4_, 0.2% dextrose) containing 0.5 mM oleic acid (OA), 0.5 mM linolenic acid (LNA), 0.5 mM vitamin E (α-tocopherol; Vit E), or 0.5mM LNA + 0.5 mM Vit E. After incubation at 37°C for 6 h, (A) cells were plated to determine CFU or (B) they were assayed for TBARS. The wild type strain was LLF100 and the Δ/Δ/Δ/Δ strain was LLF060. Error bars indicate SE. * = p<0.05, ** = p<0.01, *** = p< 0.001 by ANOVA.

To gain additional evidence that the effects of linolenic acid were due to oxidation, cells were incubated with α-tocopherol (vitamin E), a hydrophobic reducing agent that localizes to membranes and has been shown to prevent lipid peroxidation in other organisms [9, 33]. Treatment of cells with α-tocopherol alone had no detectable effects on CFUs or lipid peroxidation. In contrast, the addition of α-tocopherol significantly decreased the killing activity of linolenic acid in both WT and the Δ/Δ/Δ/Δ mutant (Fig 3A). Similarly, α-tocopherol reduced the levels of lipid peroxidation to below the limit of detection, as determined by the TBARS assay (Fig 3B).

### Heterologous expression of known NAD(P)H quinone oxidoreductases rescues the phenotypes of the Δ/Δ/Δ/Δ

**Fig 4:**
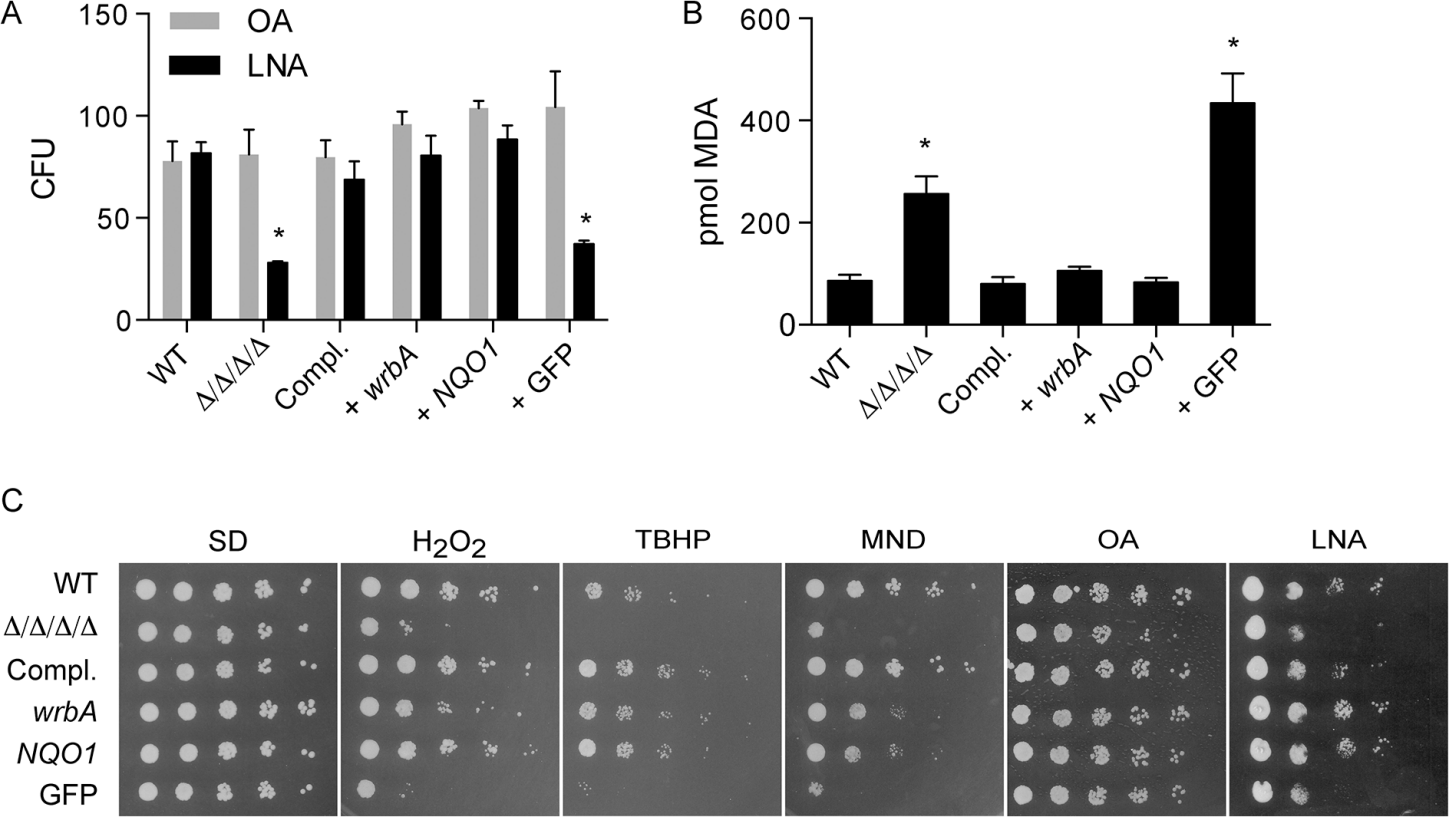
Heterologous expression of *E*. *coli wrbA* or rat *NQO1* rescues the sensitivity of Δ/Δ/Δ/Δ mutant to oxidants. The distinct NAD(P)H quinone oxidoreductase genes *wrbA* and *NQO1* were expressed in the Δ/Δ/Δ/Δ strain under control of the *ADH1* promoter. A control strain was also constructed in a similar manner that expressed GFP. The cells were then incubated with 0.5 mM linolenic acid (LNA) for 6 h at 37˚C and then (A) assayed for viable CFUs or (B) assayed for TBARS as an indicator of lipid peroxidation. Some cells in panel A were also incubated with oleic acid (OA) as a control. (C) Dilutions of cells were spotted onto different agar plates containing synthetic medium and the indicated oxidant, and then incubated at 37°C for 2 d. The plates contained H_2_O_2_, tert-butyl hydroperoxide (TBHP), cumene hydroperoxide (CHP), menadione (MND), monounsaturated oleic acid (OA), and polyunsaturated linolenic acid (LNA). Strains used included the wild type control strain LLF100, Δ/Δ/Δ/Δ strain LLF060, the complemented strain LLF079, and the Δ/Δ/Δ/Δ strain in which *E*. *coli wrbA* (LLF074), rat *NQO1* (LLF076) or GFP (LLF080) was expressed under control of the constitutive *ADH1* promoter. Error bars indicate SE. *, p <0.05 by ANOVA.

To confirm whether quinone reductase activity is important to promote resistance to oxidative stress in *C*. *albicans*, the Δ/Δ/Δ/Δ mutant was engineered to express two distinct types of NAD(P)H quinone oxidoreductases: rat *NQO1* and *E*. *coli wrbA*. *NQO1* and *wrbA* were selected because their proteins have been well-studied biochemically [21, 39, 40]. These genes were expressed under the control of the strong *ADH1* promoter. As a control, cells were also engineered to express GFP in a similar manner. Incubation of the cells in the presence of 0.5 mM linolenic acid for 6 h showed that expression of either *wrbA* or *NQO1* rescued the viability of the Δ/Δ/Δ/Δ mutant (Fig 4A). In contrast, the Δ/Δ/Δ/Δ mutant or the Δ/Δ/Δ/Δ mutant that expressed only GFP showed a significant drop in CFUs. Similarly, expression of *wrbA* or *NQO1*, but not GFP, diminished lipid peroxidation in cells that were exposed to linolenic acid (Fig 4B). Growth assays on agar plates also showed that *wrbA* and *NQO1* could complement the increased sensitivity of the Δ/Δ/Δ/Δ mutant to H_2_O_2_, tert-butyl hydroperoxide, cumene hydroperoxide and menadione (Fig 4C). The ability of two distinct quinone reductases to complement the Δ/Δ/Δ/Δ mutant phenotype demonstrates that this activity plays a key antioxidant role in *C*. *albicans*.

### Functional differences between NAD(P)H quinone oxidoreductase homologues

**Fig 5:**
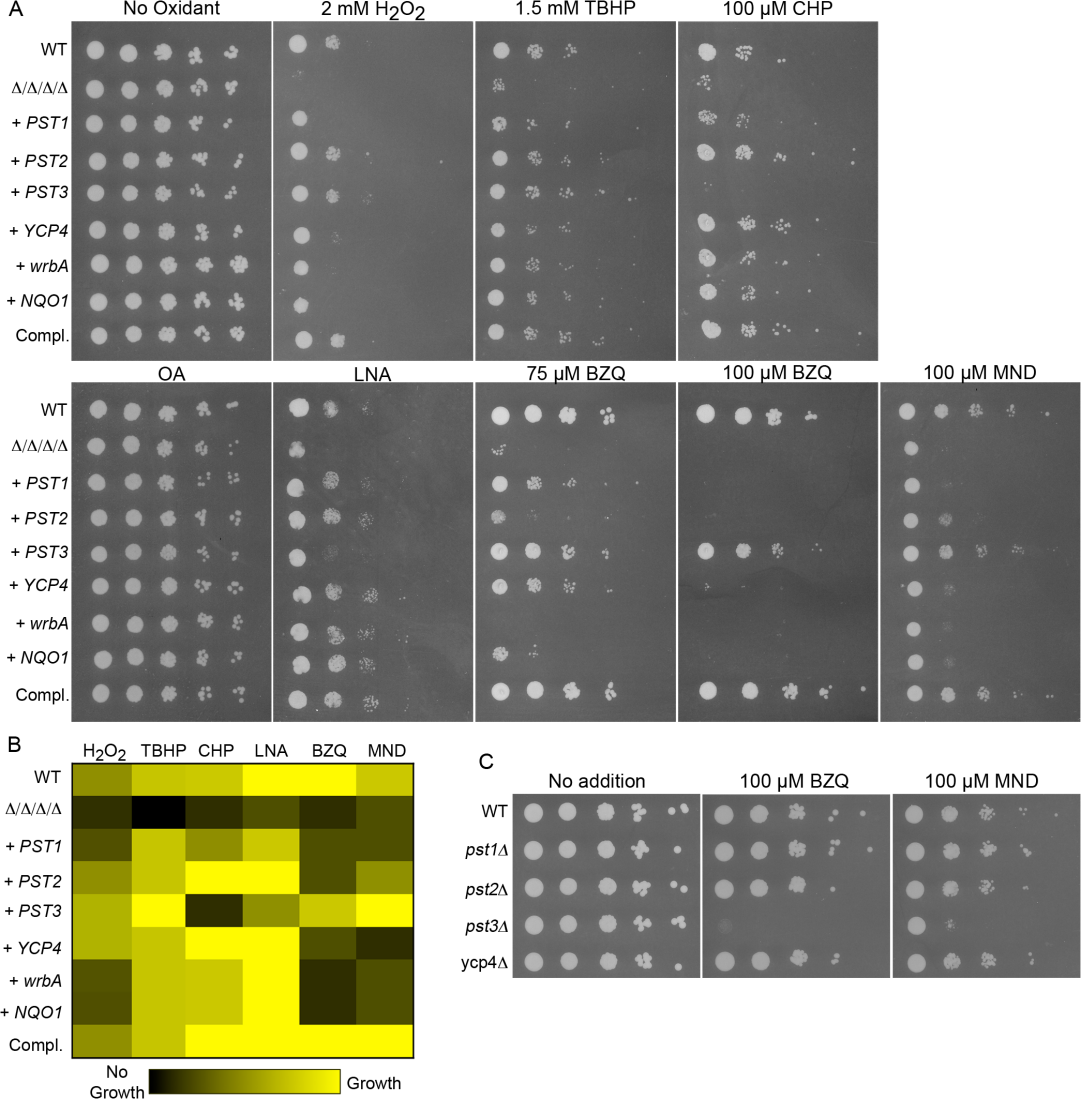
Differential sensitivity to oxidants for *C*. *albicans* strains expressing a single FLP gene or the analogous *NQO1* gene. Strains expressing individual *C*. *albicans* FLP genes under control of their native promoter, or the *E*. *coli wrbA* or rat *NQO1* gene under control of the *ADH1* promoter were created by introducing the corresponding genes into the Δ/Δ/Δ/Δ mutant strain LLF054. (A) Serial 10-fold dilutions of cells were spotted onto agar plates containing synthetic medium and the indicated chemical. Plates were incubated at 37°C for 48 h and then photographed. (B) Summary of mutant phenotypes shown in panel A. A brighter yellow color indicates better cell growth of under the specified condition. Black indicates no growth detected. The values were determined from the relative extent of growth detected in two to three independent spot assays as shown in panel A. (C) Deletion mutant strains lacking a single FLP gene were spotted onto medium containing the indicated quinones. Note that the *pst3Δ* strain was more sensitive. Strains used included the wild type control strain LLF100, Δ/Δ/Δ/Δ strain LLF060, and the complemented strain LLF079. Also, the Δ/Δ/Δ/Δ strain engineered to express *E*. *coli wrbA* (strain LLF074), rat *NQO1* (strain LLF076), *GFP* (strain LLF080), *PST1* (LLF064), *PST2* (LLF081), *PST3* (LLF066) or *YCP4* (LLF082). Single mutant deletion strains used in panel C were *pst1Δ* (LLF052), *pst2Δ* (LLF059), *pst3Δ* (LLF036), and *ycp4Δ* (LLF037).

The properties of the different quinone reductase homologues were examined by expressing individual genes in the Δ/Δ/Δ/Δ mutant. The *C*. *albicans* genes were reintroduced under control of their native promoters, whereas *wrbA* and *NQO1* were controlled by the *ADH1* promoter. Growth assays were performed to test the ability of cells carrying only one quinone reductase gene to resist different quinones and oxidants. All of the different quinone reductases were able to promote resistance to H_2_O_2_, tert-butyl hydroperoxide, and linolenic acid (Fig 5A). However, some of the strains had differential ability to resist cumene hydroperoxide and the small molecule quinones: p-benzoquinone and menadione (Fig 5A and summarized in Fig 5B).

The strain expressing only *PST3* was very interesting in that it showed the strongest resistance to p-benzoquinone and menadione (Fig 5A). Although several strains displayed obvious resistance to 75 μM p-benzoquinone, only the *PST3*-expressing strain was resistant to 100 μM p-benzoquinone. It grew remarkably better than the other strains, and nearly as well as the complemented strain that carries one copy of each FLP gene. Similarly, it also grew better than the other strains on medium containing menadione. In contrast, the *PST3*-expressing strain did not show significant resistance to cumene hydroperoxide and was more weakly resistant to linolenic acid, which are considered to be good inducers of lipid peroxidation. This strain was, however, more resistant than the Δ/Δ/Δ/Δ mutant to H_2_O_2_ and tert-butyl hydroperoxide, indicating that it can provide protection against some oxidants. Thus, it appears that Pst3 can preferentially act on small molecule quinones. In agreement with this, a *pst3Δ* strain was sensitive to the inhibitory effects of p-benzoquinone and menadione, whereas the *pst1Δ*, *pst2Δ* and *ycp4Δ* mutants were not (Fig 5C). The increased sensitivity of the *pst3Δ* mutant to p-benzoquinone and menadione were the only phenotypes we detected for the single mutants as we did not detect increased sensitivity to oxidizing conditions (Fig 2).

Some of the other strains expressing a single quinone reductase showed the opposite phenotype of being more resistant to oxidants than to the small molecule quinones. For example, the strains expressing *PST2*, *YCP4*, *wrbA*, or *NQO1* all showed improved resistance to cumene hydroperoxide and linolenic acid compared to the Δ/Δ/Δ/Δ mutant, but were not significantly more resistant or were more weakly resistant to the small molecule quinones under the conditions tested (Fig 5A). The different phenotypes indicate that there are functional differences between the various quinone reductases.

### Ubiquinone promotes resistance to oxidative stress, but not small molecule quinones

**Fig 6:**
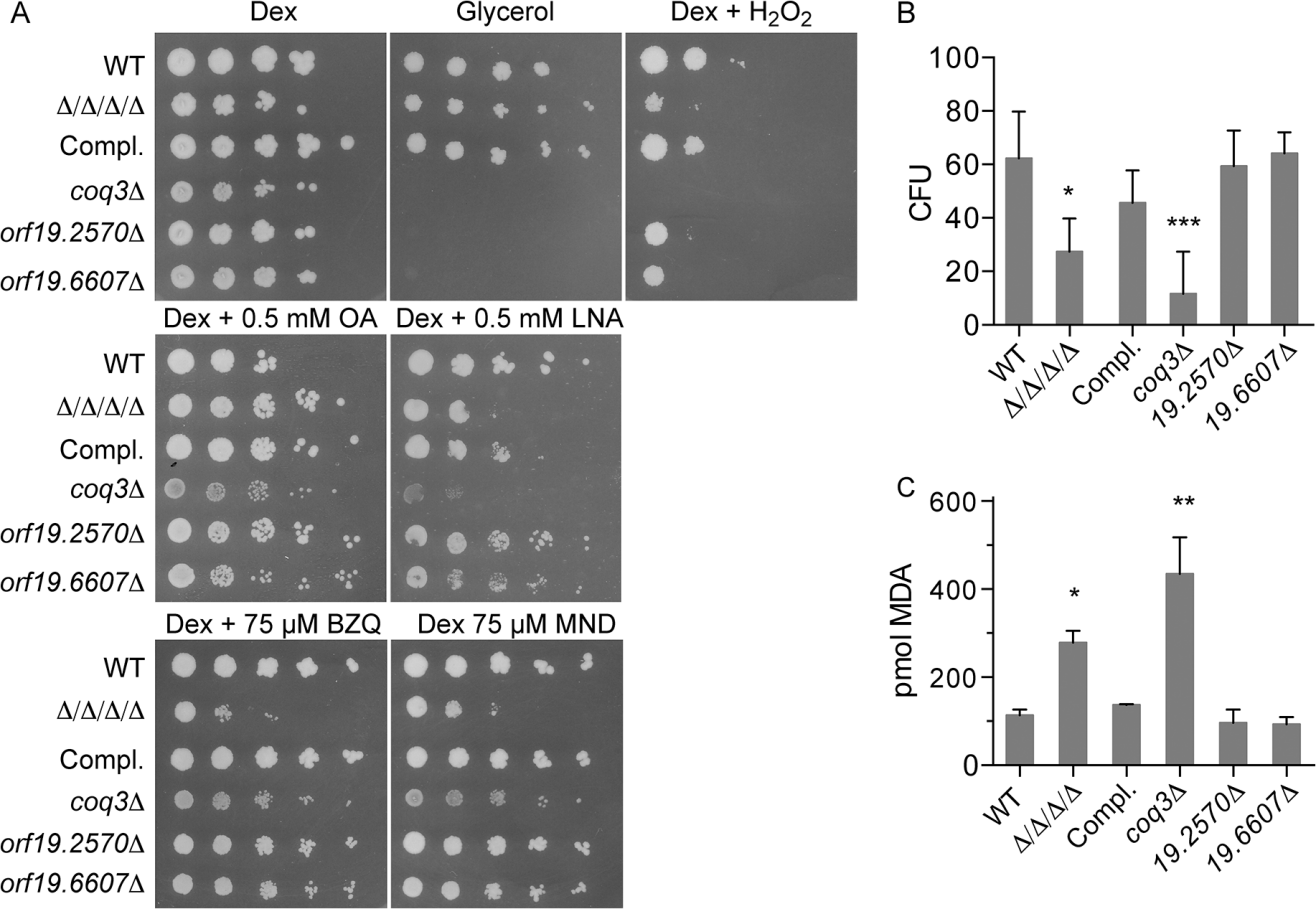
Comparison of the Δ/Δ/Δ/Δ mutant with a *coq3Δ* mutant that fails to produce ubiquinone. The **Δ/Δ/Δ/Δ** mutant was compared with a *coq3Δ* mutant (LLF088) and two representative mitochondrial mutants, *orf19*.*2570Δ*, and *orf19*.*6670Δ*. (A) Serial 10-fold dilutions of cells were spotted onto agar plates containing synthetic medium and the indicated carbon source, or with dextrose plus H_2_O_2_. Plates were incubated at 37°C for 48 h and then photographed. (B) Cells were treated for 6 hours in the presence of 0.5mM LNA, and then assayed for viable CFUs, or (C) assayed for TBARS as an indicator of lipid peroxidation. Error bars indicate SE.

The major quinone found in cells, ubiquinone (coenzyme Q), is known to have two key functions. It plays a central role in the mitochondrial electron transport chain, and it is also present in other cellular membranes where it can function as an antioxidant [30–34]. To investigate the relationship between ubiquinone and oxidative stress, both copies of *COQ3* were deleted from *C*. *albicans* to prevent ubiquinone synthesis. As expected, a *C*. *albicans coq3Δ* mutant was not able to grow on glycerol, a carbon source that requires respiration to be utilized (Fig 6A). In contrast, the Δ/Δ/Δ/Δ mutant readily grew on glycerol (Fig 6A). Interestingly, the *coq3Δ* mutant was very sensitive to H_2_O_2_, even more so than the Δ/Δ/Δ/Δ mutant (Fig 6A). Spot assays also showed that the *coq3Δ* mutant was more sensitive to linolenic acid than the Δ/Δ/Δ/Δ mutant. For comparison, two previously constructed mitochondrial mutants were examined that lack components of Complex I of the electron transport chain [41]. Both *orf19*.*2570Δ* and *orf19*.*6607Δ* failed to grow on glycerol medium, as expected (Fig 6A). However, they were not more sensitive to linolenic acid and showed perhaps only a minor increase in sensitivity to H_2_O_2_. This indicates that a mitochondrial defect does not account for the increased sensitivity to oxidation of the *coq3Δ* mutant, consistent with ubiquinone also playing a major role as an antioxidant.

Analysis of cell viability after incubation with 0.5 mM linolenic acid for 6 h revealed a larger drop in CFUs for the *coq3Δ* mutant than for the Δ/Δ/Δ/Δ mutant (Fig 6B). The *coq3Δ* mutant also displayed significantly higher levels of TBARS under these conditions (Fig 6C). Similar results have been observed in *S*. *cerevisia*e, as a *coq3Δ* mutant in this yeast is also sensitive to oxidation and lipid peroxidation [33]. These results demonstrate that ubiquinone plays an important role as an antioxidant to prevent lipid peroxidation and oxidative stress in *C*. *albicans*.

It is noteworthy that the *coq3Δ* mutant was not significantly more sensitive to p-benzoquinone and menadione, even though it was very sensitive to H_2_O_2_ and linolenic acid (Fig 6A). This suggests that the FLPs in *C*. *albicans* can detoxify these small molecule quinones in the absence of ubiquinone, thereby prevent them from causing oxidative damage.

### FLPs localize to the plasma membrane in *C*. *albicans*

**Fig 7:**
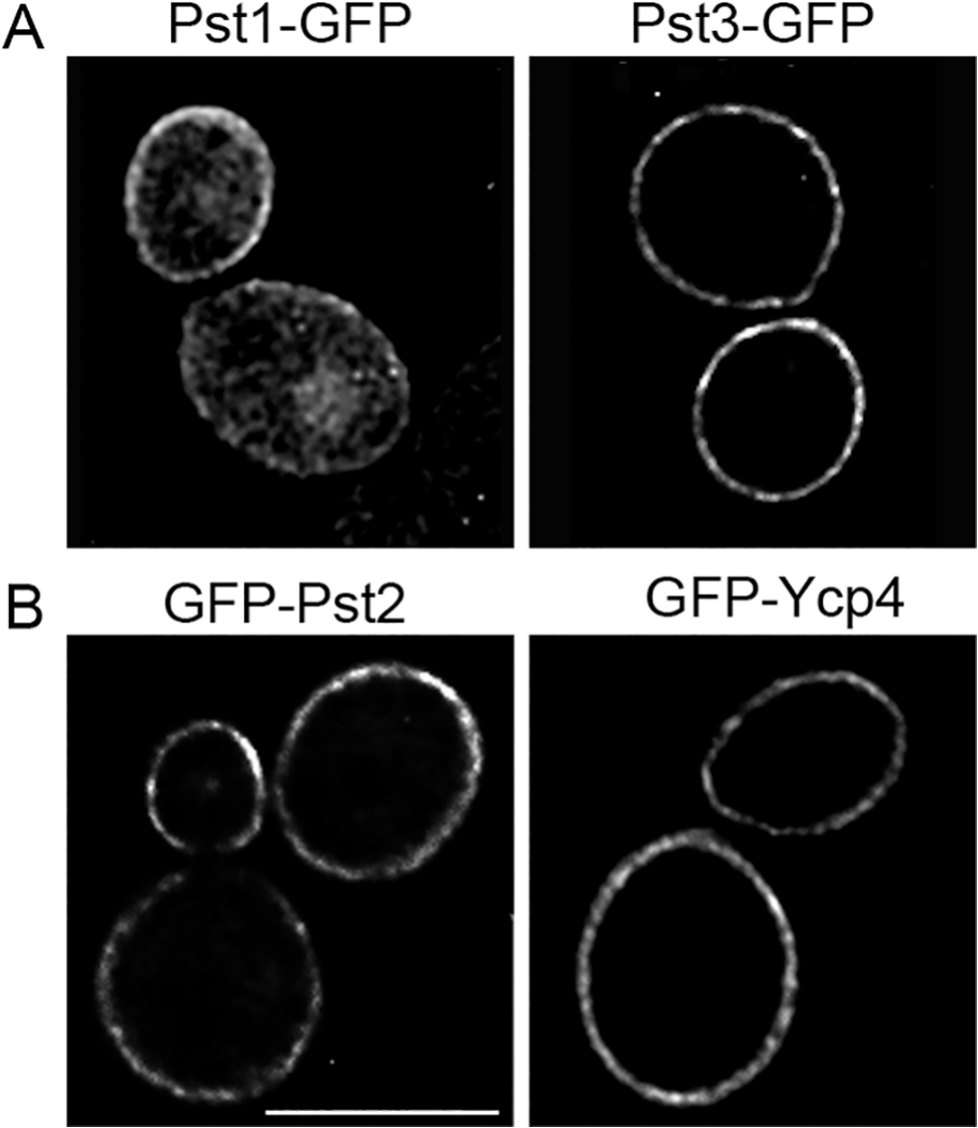
Plasma membrane localization of the *C*. *albicans* FLP proteins. Fluorescence microscopy of *C*. *albicans* cells producing (A) Pst1-GFP and Pst3-GFP fusion proteins in which GFP was added to the C terminus. (B) Cells producing GFP-Pst2 and GFP-Ycp4 fusions in which GFP was added to the N terminus. The GFP fusion genes in panel A were regulated by their endogenous promoters, whereas the fusion genes in panel B were expressed using the *ADH1* promoter.

FLPs were fused to GFP to examine their subcellular localization. Pst1-GFP and Pst3-GFP were detected at the plasma membrane by fluorescence microscopy (Fig 7A). To improve detection for the other two FLPs, the strong *ADH1* promoter was used to express GFP fusions to the *PST2* and *YCP4* genes. These GFP-Pst2 and GFP-Ycp4 fusion proteins gave a strong plasma membrane signal (Fig 7B). The GFP-tagged FLPs all showed a slightly patchy distribution in the plasma membrane, suggesting that they localize in part to the eisosome subdomains, as do their *S*. *cerevisiae* orthologs [18, 42]. Cytoplasmic GFP signal was also detected in cells. However, this could be due to proteolytic cleavage of the FLP proteins resulting in the presence of free cytoplasmic GFP, as Western blot analysis detected a strong signal at the expected size of GFP (~30 kD) (S3 Fig).

### The FLP genes promote *C*. *albicans* virulence in mice

**Fig 8:**
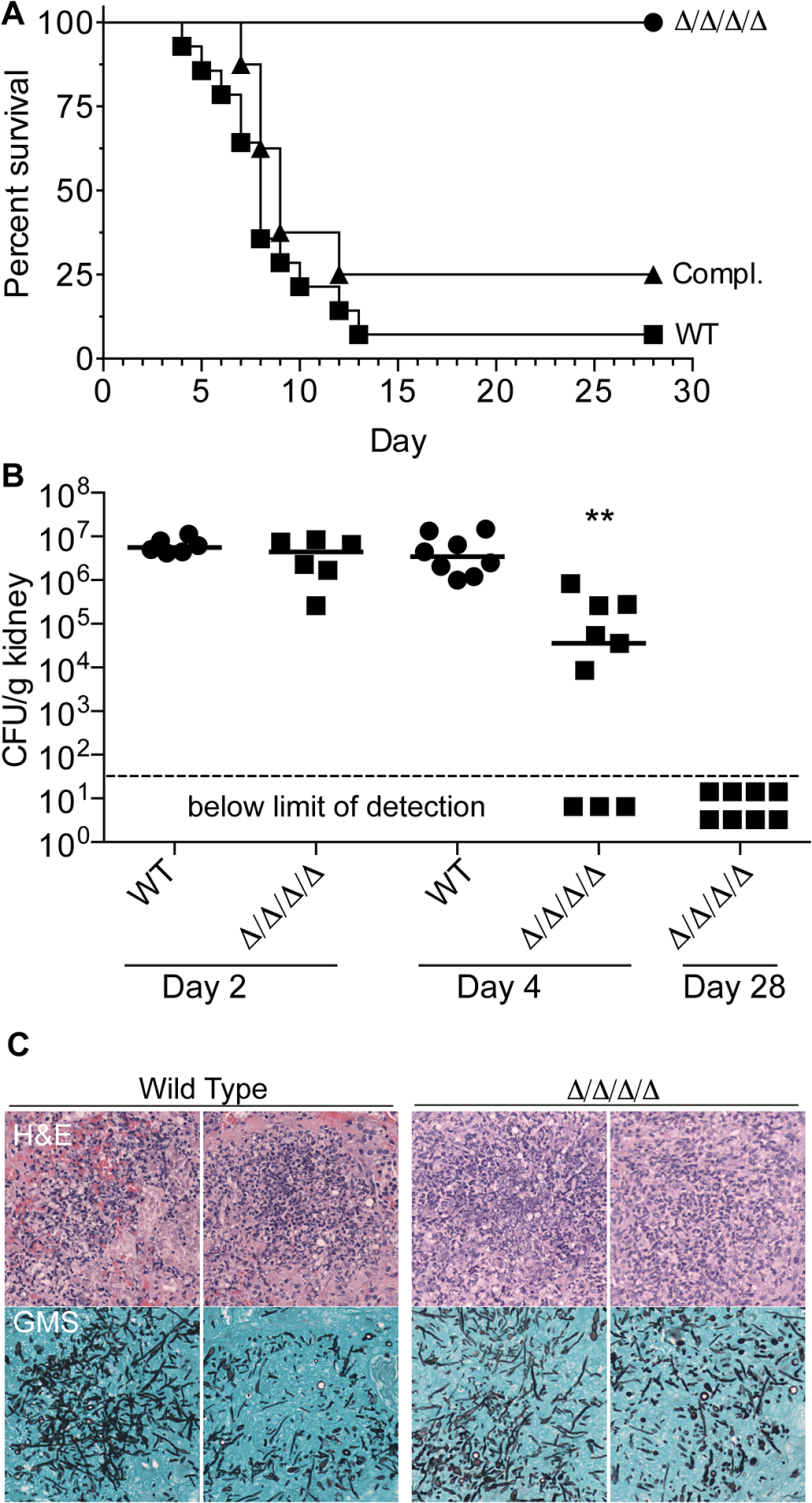
The Δ/Δ/Δ/Δ mutant is avirulent in a mouse model of systemic candidiasis. (A) 2.5 x 10^5^ *C albicans* cells were injected via the tail vein into 8-week old female Balb/c mice. Fourteen mice were injected with the wild type control strain LLF100, and 8 mice each were injected with either the Δ/Δ/Δ/Δ strain LLF060 or the complemented strain LLF079. The mice were then monitored for survival over the next 28 d. (B) Mice were infected as described above and then CFU per g kidney was determined at day 2, 4 or 28 post infection. **, p < 0.01 by ANOVA. Six mice were infected with each strain for the day 2 analysis, 8 wild type control and 9 Δ/Δ/Δ/Δ mice were infected for the day 4 analysis, and 8 mice were infected with the Δ/Δ/Δ/Δ strain for the day 28 analysis (which correspond the same mice shown in panel A). (C) Histological analysis of kidneys harvested from mice 2 d after infection with 2.5 x 10^5^ of wild type control of Δ/Δ/Δ/Δ mutant C. albicans cells. Upper panels show representative areas of the kidney stained with Hematoxylin and Eosin (H&E) to detect leukocytes (stained dark blue). Lower panels were stained with Gomori-methenamine silver (GMS) to detect fungal cells (black).

The role of the FLPs in virulence was examined using a mouse model of hematogenously disseminated candidiasis [43]. After injection via the tail vein with 2.5 x 10^5^ *C*. *albicans* cells, BALB/c mice infected with the wild type control strain succumbed to infection with a median time of 8 days (Fig 8A). Similar results were observed for the complemented version of the Δ/Δ/Δ/Δ strain. In contrast, all mice infected with the Δ/Δ/Δ/Δ mutant survived to the end of the experiment (Day 28). No CFUs were detected in the kidneys from these mice, indicating that they had cleared the infection (Fig 8B).

To determine whether the Δ/Δ/Δ/Δ mutant failed to initiate an infection, or if it was cleared more rapidly, kidneys were examined at early times post infection. The kidney is a sensitive organ to test the ability of *C*. *albicans* to initiate an infection, since this fungus grows rapidly in the kidneys during the first two days after infection [44, 45]. At day 2 post infection, the wild type and Δ/Δ/Δ/Δ mutant were both present at similarly high levels of CFU/g kidney, indicating they grew well initially (Fig 8B). Histological analysis showed that foci of *C*. *albicans* growth in the kidney overlapped with clusters of leukocytes (Fig 8C). However, by the 4th day post infection, the median CFU/g kidney was 100-fold lower for mice infected with the Δ/Δ/Δ/Δ mutant than the wild type. Furthermore, 33% of the mice (3/9) had no detectable CFU/g kidney at day 4, indicating that they had cleared the infection. Thus, the FLPs are required for the persistence of *C*. *albicans* systemic infection.

Previous studies have shown that oxidation sensitive mutants, including those with defects in catalase, thioredoxin, or superoxide dismutatase, show normal or only slightly increased sensitivity to killing by neutrophils [46]. Similar results were obtained when the Δ/Δ/Δ/Δ mutant was examined for sensitivity to killing by macrophages derived from mouse bone marrow cells. Although the Δ/Δ/Δ/Δ mutant showed a slight increase in killing by macrophages, the difference was not statistically significant (S4 Fig).

Analysis of the *pst3Δ ycp4Δ* double mutant and the *pst2Δ pst3Δ ycp4Δ* triple mutant showed that they did not display a significant virulence defect in mice (S5 Fig). Mice infected with the triple mutant appeared to show slightly increased survival (median 12.5 days) compared to the wild type control strain (median 8 days), but this difference was not statistically significant using a log rank test (Mantel-Haenszel). These results are consistent with the general redundancy of the FLP genes seen in the in vitro studies. In addition, since both the double and triple mutant lack *PST3*, this indicates that the special role this FLP plays in detoxifying small quinones does not appear to be important in systemic candidiasis.

## Discussion

Cells utilize a variety of pathways to protect against oxidation [1, 3, 5, 6]. Cytoplasmic mechanisms include superoxide dismutase, catalase, thioredoxin, and glutathione. In addition, pathogens have also evolved extracellular mechanisms. For example, *C*. *albicans* produces three superoxide dismutases that are GPI-anchored and therefore on the cell surface or built into the cell wall (Sod4-6) [5, 6]. One of these, Sod5, was recently shown to have unique properties in that it uses copper as a co-factor, but not zinc [47]. This appears to be designed to take advantage of the fact that copper is pumped into phagosomes but zinc is restricted as part of the antimicrobial attack by leukocytes. However, it is not as well understood how cellular membranes are protected from oxidation, particularly the fungal plasma membrane that is directly exposed to the oxidative attack by neutrophils and macrophages [2].

To better understand how the plasma membrane is protected against oxidation we examined four FLPs in *C*. *albicans* that are associated with the plasma membrane (Fig 7). In agreement with their predicted role as NAD(P)H quinone oxidoreductases, a *C*. *albicans* Δ/Δ/Δ/Δ quadruple mutant lacking all four FLP genes (*PST1*, *PST2*, *PST3*, and *YCP4*) displayed increased sensitivity to p-benzoquinone and menadione, a napthoquinone (Fig 1). Interestingly, the mutant cells were also more sensitive to a wide range of oxidants, indicating that they have a broader antioxidant function.

Consistent with the membrane localization of the FLPs, the Δ/Δ/Δ/Δ mutant was very sensitive to hydrophobic oxidants, including linolenic acid (Figs 1 and 5). The increased sensitivity to linolenic acid was particularly significant, since previous studies demonstrated that this PUFA auto-oxidizes and initiates a chain reaction of lipid peroxidation [33]. In agreement with this, the Δ/Δ/Δ/Δ mutant showed elevated levels of TBARS (Fig 2), a hallmark of lipid peroxidation [9, 10]. Furthermore, the effects of linolenic acid could be reversed by the hydrophobic antioxidant α-tocopherol (Vitamin E) (Fig 3). Lipid peroxidation is likely to be a more serious problem for *C*. *albicans* than for *S*. *cerevisiae*, which lacks significant levels of PUFAs [33]. About 30% of the fatty acids in *C*. *albicans* are polyunsaturated [7, 8], which predisposes them to forming lipid peroxides [9, 10]. These PUFAs are typically found in more complex lipids, such as phospholipids, in addition to existing as free fatty acids. Taken together, the results identify FLPs as an important new set of antioxidant enzymes in *C*. *albicans*. These results also have broad significance for other pathogens, given that FLPs are induced by oxidative stress in diverse fungi [11, 14, 48–50] and there is suggestive evidence that they play an antioxidant role in bacteria [17, 19, 21, 27].

### NAD(P)H quinone oxidoreductases are critical for resisting oxidative stress

Biochemical studies of FLPs from bacteria, fungi, and plants have shown that they use NAD(P)H to reduce quinones in a manner that avoids creation of hazardous semiquinone intermediates [23–27]. The Δ/Δ/Δ/Δ mutant was rescued by expression of *E*. *coli wrbA* (Fig 4), confirming that NAD(P)H quinone oxidoreductase activity plays an important antioxidant role in *C*. *albicans*. Furthermore, heterologous expression of mammalian *NQO1* in the Δ/Δ/Δ/Δ mutant also rescued its sensitivity to oxidation and lipid peroxidation. Nqo1 does not share obvious sequence similarity with FLPs even though it carries out a similar enzymatic activity. Although there are some underlying structural similarities between Nqo1 and FLPs, they are quite distinct [19]. For example, Nqo1 binds FAD as a cofactor instead of FMN, and it forms dimers rather than tetramers as seen for wrbA. These observations provide strong support that the key function of the *C*. *albicans* FLPs is to act as quinone reductases.

Analysis of Δ/Δ/Δ/Δ cells engineered to express a single FLP gene indicated that they have overlapping but distinct functions. Pst3 provided the best protection against the small molecule quinones p-benzoquinone and menadione (Fig 5). In agreement with this, a *pst3Δ* mutant was the only single FLP deletion mutant that was more sensitive to the small molecule quinones (p-benzoquinone and menadione) (Fig 5). In contrast, cells expressing only *PST3* were less able to resist other oxidants, such as linolenic acid or cumene hydroperoxide. These phenotypes are consistent with different functional properties. However, it is also possible that some of these differences are due to differential expression of the various FLP genes under the different conditions that were tested.

### Ubiquinol is an important antioxidant in *C*. *albicans*

The most likely target for the quinone reductase activity of FLPs in *C*. *albicans* is ubiquinone (coenzyme Q). Ubiquinone has a benzoquinone head group and a hydrophobic isoprenylated tail that localizes it to membranes [32, 51]. Analogous to its well-known role as an electron carrier in the mitochondria, ubiquinone is present in other cellular membranes where its reduced form (ubiquinol) can act as an antioxidant [30–34]. In particular, ubiquinol is thought to be able to reduce lipid radicals that would otherwise propagate a chain reaction of lipid peroxidation to cause more extensive damage [9, 10]. To determine if ubiquinol plays an important antioxidant role in *C*. *albicans*, *COQ3* was deleted to block its synthesis. The *coq3Δ* mutant was found to be very sensitive to oxidative stress and also displayed increased levels of lipid peroxidation in response to linolenic acid (Fig 6). In further support of the conclusion that FLPs act on ubiquinone, rat *NQO1*, which is known to reduce ubiquinone [35, 36], can rescue the defects of the Δ/Δ/Δ/Δ mutant (Figs 4 and 5).

There were interesting differences between the Δ/Δ/Δ/Δ mutant and the *coq3Δ* mutant that reveal insights into their roles. Whereas the *coq3Δ* mutant was highly sensitive to oxidizing conditions promoted by peroxides or PUFAs, it was not significantly altered in sensitivity to p-benzoquinone and menadione (Fig 6). This indicates that the FLPs can reduce quinones in the absence of ubiquinol. The *coq3Δ* mutant was also much more sensitive than the Δ/Δ/Δ/Δ mutant to H_2_O_2_ and linolenic acid. One possibility is that other reductases can contribute to reduction of ubiquinone in the absence of the FLPs. However, if these enzymes use a one-electron mechanism for reduction of quinones, they will generate deleterious semiquinone radicals that would contribute to the phenotype of the Δ/Δ/Δ/Δ mutant.

### FLPs are required for virulence and represent novel drug targets

The FLPs were required for virulence in a mouse model of hematogenously disseminated candidiasis (Fig 8A). Whereas the median survival time was 8 days for mice injected with 2.5 x 10^5^ wild type *C*. *albicans*, all of the mice infected with the Δ/Δ/Δ/Δ mutant survived to the end of the experiment (day 28). Thus, the Δ/Δ/Δ/Δ mutant appears to have a stronger virulence defect than was reported for other *C*. *albicans* oxidation sensitive mutants including a *cat1Δ* catalase mutant [52], a *sod1Δ* or *sod5Δ* superoxide dismutase mutant [53, 54], a *grx2Δ* glutathione reductase mutant [53], or a *tsa1Δ* thioredoxin peroxidase mutant [55].

Interestingly, the Δ/Δ/Δ/Δ mutant could initially grow in the kidney essentially as well as a wild type strain (Fig 8B). However, by day 4 there was about a 100-fold decrease in median CFUs and 3 out of 9 mice cleared the infection. This decline in CFUs for the Δ/Δ/Δ/Δ mutant correlates with the influx of neutrophils (Fig 8C) that typically peaks about day 2 of infection [44, 45]. By day 28, all of the mice infected with the Δ/Δ/Δ/Δ mutant lacked detectable CFU and appear to have cleared the infection. Generally similar results were reported for a *C*. *albicans cat1Δ* catalase mutant that also grew well initially, but then CFUs declined in most infected mice [52]. In this regard it is also significant that a *wrbAΔ* mutant of the bacterial pathogen *Yersinia tuberculosis* can initiate an infection but is defective in establishing a persistent infection [56].

This key role in virulence for the FLPs indicates they have strong potential to serve as novel targets for antifungal therapy. New therapeutic approaches are needed; ~40% of patients with systemic candidiasis succumb to the infection even with current antifungal therapy [57, 58]. This outcome is likely to worsen, as drug resistance is a growing problem for two of the three most commonly used antifungal drugs [59, 60]. An important advantage of targeting FLPs is that they do not have orthologs in humans. The analogous NAD(P)H quinone oxidoreductases in mammals, Nqo1 and Nqo2, are very different [19].

Pharmacological studies on Nqo1 have identified multiple ways that quinone reductases can be targeted. One approach is to identify inhibitors, such as dicoumarol that blocks the Nqo1 activity [61]. In addition, the ability of Nqo1 to reduce small molecule quinones has been studied as a basis for cancer chemotherapy. The fact that many cancer cells overexpress *NQO1* has been exploited to develop novel therapies in which quinone compounds are reduced by Nqo1 to convert them into a toxic form that preferentially kills cancer cells [36, 62]. Similarly, Nqo1 has also been shown to reduce benzoquinone-containing ansamycin drugs, which makes these compounds more potent inhibitors of the Hsp90 chaperone [63]. This suggests yet another way drugs targeting FLPs could be useful, since Hsp90 inhibitors can prevent the emergence of drug resistance in *C*. *albicans* [64]. Thus, the important roles of FLPs in oxidative stress response and virulence, combined with their absence in mammalian cells, identifies them as important new targets for therapeutic strategies aimed at combating fungal and bacterial pathogens.

## Materials and Methods

### Ethics statement

All procedures were approved by the Stony Brook University IACUC Committee (#1686). Mice were considered to be moribund if food and water could no longer be accessed and then humane euthanasia was performed by carbon dioxide inhalation as per instructions from the Department of Laboratory Animals at Stony Brook University.

### Chemicals, strains and media

Oleic acid, linoleic acid, linolenic acid, α-tocopherol (vitamin E), hydrogen peroxide, tert-butyl hydroperoxide, cumene hydroperoxide, menadione, p-benzoquinone, thiobarbituric acid (TBA), hydrochloric acid, and 1,1,3,3-tetramethoxypropane were purchased from Sigma-Aldrich Corp. Trichloroacetic acid was from the Alfa Aesar Company, and nourseothricin from Werner BioAgents.

The *C*. *albicans* strains used in this study are described in Table 1. Cells were grown in SD medium (yeast nitrogen base synthetic medium with dextrose) [65]. *C*. *albicans* deletion mutants were created in strain SN152 (*arg4Δ his1Δ leu2Δ*) by homologous recombination, as described previously [66]. Mutant strains that carry homozygous deletion of *PST1*, *PST2*, *PST3*, *YCP4*, *or COQ3* were constructed with strain SN152 by the sequential deletion of both copies of the targeted gene. Gene deletion cassettes were generated by PCR amplification of the *LEU2* or *HIS1* selectable marker gene [66], using primers that also included ~80 bp of DNA sequence homologous to the upstream or downstream region of the targeted open reading frame (ORF). Cells that had undergone homologous recombination to delete the targeted gene were identified by PCR analysis. A *pst3Δ ycp4Δ* double mutant strain was constructed by simultaneous deletion of both genes, taking advantage of the fact that they are adjacent in the genome. Homozygous triple and quadruple deletion mutation strains were then constructed by sequential deletion of both copies of the targeted gene using the *SAT1* flipper method to recycle the selectable marker [37]. Similar phenotypes were observed for independent isolates. Deletion strains were then made prototrophic by transforming with the *ARG4* gene to correct the remaining auxotrophy.

**Table 1:** Strains used.

*C*. *albicans* strain	Parent	Genotype
SN152	SC5314	*arg4Δ/arg4Δ leu2Δ/leu2Δ his1Δ/his1Δ URA3/ura3Δ*::*imm IRO1/iro1Δ*::*imm*
LLF100	SN152	(prototrophic wild type control) *arg4Δ/ARG4 leu2Δ/LEU2 his1Δ/HIS1 URA3/ura3Δ*::*imm IRO1/iro1Δ*::*imm*
LLF052	SN152	(*pst1Δ*) *pst1Δ*::*HIS1/pst1Δ*::*LEU2 ARG4/arg4Δ*
LLF059	SN152	(*pst2Δ*) *pst2Δ*::*HIS1/pst2Δ*::*LEU2 ARG4/arg4Δ*
LLF036	SN *pst3Δ*	(*pst3Δ*) *pst3Δ*::*HIS1/pst3Δ*::*LEU2 ARG4/arg4Δ*
LLF037	SN *ycp4Δ*	(ycp4Δ) *ycp4Δ*::*HIS1/ycp4Δ*::*LEU2 ARG4/arg4Δ*
LLF025	SN152	(*pst3Δ ycp4Δ*) *pst3-ycp4Δ*::*HIS1/pst3-ycp4Δ*::*LEU2 arg4Δ/arg4Δ*
LLF034	LLF025	(*pst3Δ ycp4Δ*) *pst3-ycp4Δ*::*HIS1/pst3-ycp4Δ*::*LEU2 ARG4/arg4Δ*
LLF032	LLF025	*(pst2Δ pst3Δ ycp4Δ) pst3-ycp4Δ*::*HIS1/pst3-ycp4Δ*::*LEU2 pst2Δ*::*frt/pst2Δ*::*frt arg4Δ/arg4Δ*
LLF063	LLF032	*(pst2Δ pst3Δ ycp4Δ) pst3-ycp4Δ*::*HIS1/pst3-ycp4Δ*::*LEU2 pst2Δ*::*frt/pst2Δ*::*frt ARG4/arg4Δ*
LLF054	LLF032	(Δ/Δ/Δ/Δ) *pst3-ycp4Δ*::*HIS1/pst3-ycp4Δ*::*LEU2 pst2Δ*::*frt/pst2Δ*::*frt pst1Δ*::*frt/pst1Δ*::*frt arg4Δ/arg4Δ*
LLF060	LLF054	(Δ/Δ/Δ/Δ) *LLF054 except for ARG4/arg4Δ*
LLF079	LLF054	(Compl.) *pst3-ycp4Δ*::*HIS1/pst3-ycp4Δ*::*LEU2 pst2Δ*::*frt/pst2Δ*::*frt pst1Δ*::*frt/pst1Δ*::*frt PST2-PST1*::*ARG4 arg4Δ/arg4Δ PST3-YCP4*::*SAT1*
LLF074	LLF054	(+*wrbA*) *pst3-ycp4Δ*::*HIS1/pst3-ycp4Δ*::*LEU2 pst2Δ*::*frt/pst2Δ*::*frt pst1Δ*::*frt/pst1Δ*::*frt pADH1-GFPᵧ-EcWrbA*::*ARG4 arg4Δ/arg4Δ*
LLF076	LLF054	(+*NQO1*) *pst3-ycp4Δ*::*HIS1/pst3-ycp4Δ*::*LEU2 pst2Δ*::*frt/pst2Δ*::*frt pst1Δ*::*frt/pst1Δ*::*frt pADH1-GFPᵧ-rat NQO1*::*ARG4 arg4Δ/arg4Δ*
LLF080	LLF054	(+GFP) *pst3-ycp4Δ*::*HIS1/pst3-ycp4Δ*::*LEU2 pst2Δ*::*frt/pst2Δ*::*frt pst1Δ*::*frt/pst1Δ*::*frt pADH1-GFPᵧ*::*ARG4 arg4Δ/arg4Δ*
LLF064	LLF054	(+*PST1*) *pst3-ycp4Δ*::*HIS1/pst3-ycp4Δ*::*LEU2 pst2Δ*::*frt/pst2Δ*::*frt pst1Δ*::*frt/pst1Δ*::*frt PST1*::*ARG4 arg4Δ/arg4Δ*
LLF081	LLF054	(+*PST2*) *pst3-ycp4Δ*::*HIS1/pst3-ycp4Δ*::*LEU2 pst2Δ*::*frt/pst2Δ*::*frt pst1Δ*::*frt/pst1Δ*::*frt PST2*::*ARG4 arg4Δ/arg4Δ*
LLF066	LLF054	(+*PST3*) *pst3-ycp4Δ*::*HIS1/pst3-ycp4Δ*::*LEU2 pst2Δ*::*frt/pst2Δ*::*frt pst1Δ*::*frt/pst1Δ*::*frt PST3*::*ARG4 arg4Δ/arg4Δ*
LLF082	LLF054	(+*YCP4*) *pst3-ycp4Δ*::*HIS1/pst3-ycp4Δ*::*LEU2 pst2Δ*::*frt/pst2Δ*::*frt pst1Δ*::*frt/pst1Δ*::*frt YCP4*::*ARG4 arg4Δ/arg4Δ*
LLF083	SN orf19.2570Δ	*orf19*.*2570Δ*::*HIS1/ orf 19*.*2570Δ*::*LEU2 ARG4/arg4Δ*
LLF084	SN orf19.6607Δ	*orf19*.*6607Δ*::*HIS1/ orf 19*.*6607Δ*::*LEU2 ARG4/arg4Δ*
LLF088	SN152	(*coq3Δ*) *coq3Δ*::*HIS1/coq3Δ*::*LEU2 ARG4/arg4Δ*
BWP17	SC5314	*ura3*Δ::λ*imm434/ura3* Δ::λ *imm434 his1*::*hisG/his1*::*hisG arg4*::*hisG/arg4*::*hisG*
LLF018	BWP17	*LSP1/LSP1-mCherry*::*ARG4 ura3*Δ::λ*imm434/ura3* Δ::λ *imm434 his1*::*hisG/his1*::*hisG arg4*::*hisG/arg4*::*hisG*
LLF098	LLF018	*PST1/PST1-GFPγ*::*HIS1 LSP1/LSP1-mCherry*::*ARG4 ura3*Δ::λ*imm434/ura3* Δ::λ *imm434 his1*::*hisG/his1*::*hisG arg4*::*hisG/arg4*::*hisG*
LLF090	LLF018	*PST3/PST3-GFPγ*::*HIS1 LSP1/LSP1-mCherry*::*ARG4 ura3*Δ::λ*imm434/ura3* Δ::λ *imm434 his1*::*hisG/his1*::*hisG arg4*::*hisG/arg4*::*hisG*
LLF071	LLF018	*pADH1-GFPγ-YCP4*::*URA3 LSP1/LSP1-mCherry*::*ARG4 ura3*Δ::λ*imm434/ura3* Δ::λ *imm434 his1*::*hisG/his1*::*hisG arg4*::*hisG/arg4*::*hisG*
LLF089	BWP17	*pADH1-mCherry-YCP4*::*SAT1 ura3*Δ::λ*imm434/ura3* Δ::λ *imm434 his1*::*hisG/his1*::*hisG arg4*::*hisG/arg4*::*hisG*
LLF092	LLF089	*pADH1-GFPγ-PST2*::*URA3 pADH1-mCherry-YCP4*::*SAT1 ura3*Δ::λ*imm434/ura3* Δ::λ *imm434 his1*::*hisG/his1*::*hisG arg4*::*hisG/arg4*::*hisG*

Complemented strains, in which the wild-type FLP gene was reintroduced into the corresponding deletion mutant, were constructed by first using PCR to amplify the corresponding FLP gene plus 2000 base pairs (bp) upstream and 501 bp downstream of the *PST1* open reading frame (ORF), 811 bp upstream and 427 bp downstream of the *PST2* ORF, 1681 bp upstream and 427 bp downstream of the *PST3* ORF, or 1526 bp upstream and 310 bp downstream of the *YCP4* ORF. The DNA fragments were then inserted between the *Sac*I and *Sac*II sites in a derivative of plasmid pDDB57 [67] in which the *URA3* gene was replaced with *ARG4*. The resulting plasmids were then linearized by restriction digestion in the promoter region, and then transformed into the corresponding homozygous deletion mutant strains using *ARG4* for selection. The complementing plasmids were also transformed into the Δ/Δ/Δ/Δ mutant to create strains that express only a single FLP gene. A fully complemented quadruple mutant strain was constructed essentially as described above, except that both *PST1* and *PST2* genes were cloned in tandem into the *ARG4* plasmid. The plasmid was digested with *BspE*I to linearize it in the promoter region of the *PST1* gene, and then it was transformed into the quadruple mutant strain LLF054. The *PST3* and *YCP4* genes were cloned between the *Sac*I and *Apa*I restriction sites of a derivative of plasmid pDDB57 in which the *URA3* selectable marker was changed to the *SAT1* gene to confer nourseothricin resistance. Note that the *PST3* and *YCP4* genes are adjacent in the genome in a head to head manner, such that the corresponding open reading frames are transcribed in a divergent manner. A PCR fragment that contains sequences between 1157 bp downstream of the *YCP4* ORF and 466 bp downstream of the *PST3* ORF was used to create the *PST3-YCP4* complementing plasmid. The resulting plasmid was digested with *Sna*BI to linearize it about 400 bp downstream of the *YCP4* open reading frame, and then the DNA was transformed into strain LLF078, a version of the Δ/Δ/Δ/Δ quadruple mutant in which the *PST1* and *PST2* genes were already introduced as described above.

The open reading frames for *E*. *coli wrbA* and rat *NQO1* were synthesized by GeneWiz Corp. so that the codons could be optimized and to avoid CUG codons that are translated differently in *C*. *albicans*. The *wrbA* and *NQO1* open reading frames were amplified by PCR and introduced downstream of the *ADH1* promoter and GFP in plasmid pND391 that carries the *ARG4* selectable marker. The resulting plasmids were then transformed into the Δ/Δ/Δ/Δ mutant strain LLF054 to create strains expressing *wrbA* (LLF074), *NQO1* (LLF076), or GFP (LLF080) as a control.

### Growth assays to test sensitivity to oxidizing agents

Spot assays to test growth in the presence of oxidizing agents were carried out essentially as described previously [68, 69]. *C*. *albicans* mutant or wild type strains were grown overnight and then adjusted to 10^7^ cells/ml. Serial 10-fold dilutions of cells were prepared, and three μl of each dilution was then spotted onto solid agar SD medium containing the indicated chemical. The plates were incubated at 37°C for 2 days and then photographed. Each assay was done at least three independent times.

Cells were also tested in liquid culture for sensitivity to oxidizing agents by assaying colony-forming units (CFUs). *C*. *albicans* cells were grown in synthetic medium with 2% dextrose and without amino acids at 37°C overnight. Cells were harvested at about 6–10 x 10^7^ cells per ml, washed once, and resuspended in phosphate buffer (0.1M sodium phosphate, pH 6.2, 0.2% dextrose) at 10^7^ cells per ml. Three ml was transferred to a 15 ml glass test tube and then fatty acids were added. Cells were then incubated at 37°C on a tube rotator for the designated period of time. At the end of each treatment, cells were harvested by centrifugation and samples were plated to determine the number of viable CFUs. Results represent the average of 3–6 independent assays.

### Detection of thiobarbituric acid reactive substances (TBARS)

The level of TBARS in yeast whole cell lysates was determined by a modification of a previously described procedure [70]. At the end of the fatty acid treatment described above, 1.5 x 10^7^ cells were harvested by centrifugation at 17,000 x g for 5 min, washed once with distilled water, and resuspended in 100 μl distilled water in a screw cap tube. 1ml of a freshly prepared solution of 0.375% thiobarbituric acid dissolved in 12% trichloroacetic acid and 0.5 M hydrochloric acid was added to each tube. After a 20-minute incubation at 90°C, samples were allowed to cool down, and then the insoluble material was sedimented by centrifugation at 17,000 x g for 5 min. The absorbance of the supernatant was measured at 535 nm, and corrected by subtracting the nonspecific absorbance at 600 nm. The corrected absorbance was then compared with a standard curve created using 1,1,3,3-tetramethoxypropane treated under the same conditions, which generates malondialdehyde (MDA). Results represent the average of 3–4 independent experiments.

### Microscopic analysis of GFP fusion proteins

The GFPγ variant was fused to the 3’ ends of the open reading frames for *PST1* and *PST3* using *HIS1* selection, in LLF018, as described previously [71]. Strains were verified by PCR analysis and microscopic examination of GFP fluorescence. To add GFP at the 5’ end of the open reading frames to create N-terminal fusions, GFPγ was introduced downstream of the *ADH1* promoter followed by the FLP gene and then the *ADH*1 terminator in pND397, which carries an *URA3* selectable marker gene [72]. The plasmids of pADH1-GFPγ-PST1, pADH1-GFPγ-PST2, or pADH1-GFPγ-PST3 were also linearized with Not I, before being individually transformed into LLF089 using *URA3* as the selectable marker to create the strains LLF091, LLF092, and LLF093. The plasmid pADH1-GFPγ-YCP4 was linearized with *Not*I and transformed into LLF018 using *URA3* as the selectable marker to construct the strain LLF071. GFP fluorescence was analyzed directly in live cells without further processing using a Zeiss Axiovert 200M microscope equipped with an AxioCam HRm camera and Zeiss AxioVision software for deconvolving images.

### Mouse virulence assays

The survival of *C*. *albicans* cells in the presence of macrophages was assayed essentially as described previously [73]. Bone marrow was isolated from femurs of 6- to 8-week-old female C57BL/6 mice (Jackson Laboratories) and then macrophages were derived from them as previously described [74]. At 18 h prior to infection, bone marrow derived macrophages were seeded into multiwell trays in Dulbecco’s modified Eagle medium (Invitrogen) supplemented with 10% fetal bovine serum (HyClone), 15% L-cell-conditioned medium, 1 mM sodium pyruvate, 2 mM glutamate, and 100 ng/ml *E*. *coli* LPS (Sigma). Dilutions of *C*. *albicans* cells were then plated in multiwell trays in the presence or absence of the macrophages and incubated for 48 h [73]. Microcolonies of growth in each well were then counted to determine the reduction in *C*. *albicans* viability due to the presence of macrophages. The results represent the average of three different experiments in which different batches of bone marrow-derived macrophages were used.

*C*. *albicans* strains were tested for virulence in a mouse model of hematogenously disseminated candidiasis similar to previous studies [45, 75]. *C*. *albicans* cells were cultured by growing overnight at 30°C in YPD medium with 80 μg/ml uridine, reinoculating into fresh medium, and incubating again overnight at 30°C. Cells were prepared for infection assays by washing twice in phosphate-buffered saline (PBS), counting in a hemocytometer, and then diluting to 1.25 x 10^6^ cells/ml with PBS. Female BALB/c mice were injected via the lateral tail vein with 2.5 x 10^5^ cells, and then monitored at least twice a day for 28 days. Statistical analyses of the results for the survival studies were carried out using a log rank test (Mantel-Haenszel) with the Prism 6 software program (GraphPad Software, Inc., La Jolla, CA). To assess fungal burden, kidneys were excised, weighed, and then homogenized in 5 ml PBS for 30 s with a tissue homogenizer (Pro Scientific Inc.). The CFU per gram of kidney was determined by plating dilutions of the homogenates on YPD agar medium plates, and incubating for 2 days at 30°C. Statistical analysis of the CFU data was carried out with Prism 6 software using one-way analysis of variance with a nonparametric Kruskal-Wallis test and Dunn’s post-hoc test. For histological analysis, kidneys were excised from mice 2 d post infection, fixed with formaldehyde, and then stained with Hematoxylin and Eosin (H&E) to detect leukocytes or with Gomori-Methenamine Silver (GMS) to detect fungal cells by McClain Laboratories, Smithtown, NY.

### Accession numbers

The accession numbers for the *C*. *albicans* genes used in this study are as follows:

$$\begin{array}{l} \text{Standard Name}\;\text{Systematic Name}\;\text{Orf}19\;\text{Name}\;\text{GenBank Designation}\\ PST1\;\text{C}2\_06870\text{C}\;\text{orf}19.2241\;\text{XP}\_714771.1\\ PST2\;\text{C}2\_08640\text{C}\;\text{orf}19.3612\;\text{XP}\_714456.1\\ PST3\;\text{CR}\_05390\text{W}\;\text{orf}19.5285\;\text{XP}\_710366.1\\ YCP4\;\text{CR}\_05380\text{C}\;\text{orf}19.5286\;\text{XP}\_710367.1\\ COQ3\;\text{C}6\_01840\text{C}\;\text{orf}19.3400\;\text{XP}\_716710.1 \end{array}$$

## Supporting Information

S1 Fig Amino acid alignment of *C*. *albicans* FLPs and E. coli WrbA. The amino acid sequences were aligned using the Clustal W program. Key regions in the WrbA protein are highlighted, as determined by analysis of the high-resolution crystal structure of E. coli WrbA [19, 22]. Note that there is a high degree of amino acid similarity in the critical functional sites.
(TIF)

S2 Fig The Δ/Δ/Δ/Δ mutant strain is not more sensitive to oleic acid. *C*. *albicans* strains were incubated with 0.5 mM oleic acid (OA) at 37°C for the indicated time (hours), and then dilutions of cells were plated to determine the viable colony forming units (CFU). These studies assays were carried out as controls for the cells incubated in the presence of linolenic acid (LNA) shown in Fig 2A. Strains used included the wild type strain LLF100, Δ/Δ/Δ/Δ strain LLF060, and the complemented strain LLF079 in which one copy of each FLP gene was introduced into the Δ/Δ/Δ/Δ strain.
(TIF)

S3 Fig Western blot analysis of GFP fusion proteins. *C*. *albicans* cells containing fusions between GFP and the indicated FLP gene were analyzed on a Western blot probed with a mouse monoclonal anti-GFP antibody. The protein bands were detected using a secondary IRDye 800CW conjugated Goat (polyclonal) anti-mouse IgG antibody, and an image was acquired using a digital Odyssey infrared imaging system. To gain increased sensitivity, samples on the left side were expressed using a *MET3* promoter and an *ADH1* promoter was used for the samples on the right side, as indicated at the bottom. The position of pre-stained molecular weight markers (kD) is shown on the left side. The expected position for GFP is indicated on the right. Also on the right side, Pst indicates the approximate position for a GFP fusion to the Pst1, Pst2 or Pst3 proteins, and Ycp4 indicates the position for GFP-Ycp4. Note that Ycp4 is about 8 kD larger than the Pst1, Pst2, and Pst3 proteins and displays the expected difference in gel mobility on the blot.
(TIF)

S4 Fig Macrophage killing of *C*. *albicans* strains. *C*. *albicans* strains were plated in multiwell trays in the absence or presence of macrophages derived from mouse bone marrow cells. Microcolonies of growth in each well were then counted to determine the reduction in *C*. *albicans* viability due to the presence of macrophages [73]. The results represent the average of three independent experiments. There was no significant difference in killing of the different *C*. *albicans* strains, as determined by ANOVA. Error bars indicate SD.
(TIF)

S5 Fig Mouse virulence assays for the double mutant (*pst3Δ ycp4Δ*) and triple mutant (*pst2Δ pst3Δ ycp4Δ*). The double (*pst3Δ ycp4Δ*) and triple (*pst2Δ pst3Δ ycp4Δ*) FLP mutant *C*. *albicans* strains were assayed for virulence in a mouse model of systemic Candidiasis as described in Fig 8. Although mice infected with the triple mutant appeared to show slightly better survival, it was not statistically significant as judged by a log rank test (Mantel-Haenszel). Wild type control strain LLF100 was used to infect 14 mice, *pst3Δ ycp4Δ* strain LLF034 was used to infect 7 mice, and *pst2Δ pst3Δ ycp4Δ* strain LLF063 was used to infect 8 mice.
(TIF)
