# Supplementary figures and images for: Flavodoxin-Like Proteins Protect Candida albicans from Oxidative Stress and Promote Virulence
Source: PLoS Pathog. 2015 Sep 1;11(9):e1005147. doi: 10.1371/journal.ppat.1005147 (PMC4556627; doi:10.1371/journal.ppat.1005147)

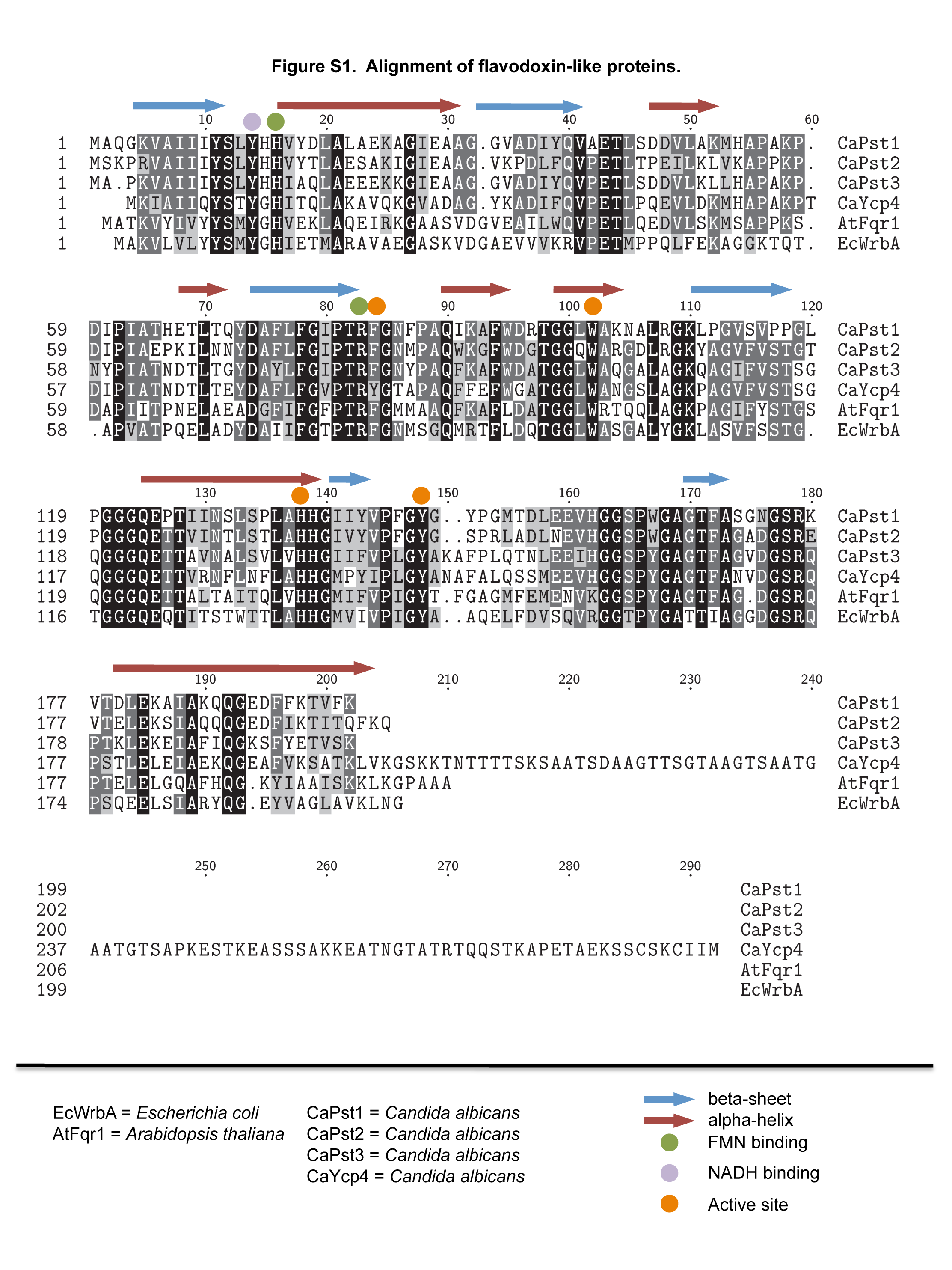

Supplement: S1 Fig — The amino acid sequences were aligned using the Clustal W program. Key regions in the WrbA protein are highlighted, as determined by analysis of the high-resolution crystal structure of E. coli WrbA [19, 22]. Note that there is a high degree of amino acid similarity in the critical functional sites. (TIF) [file ppat.1005147.s001.tif]

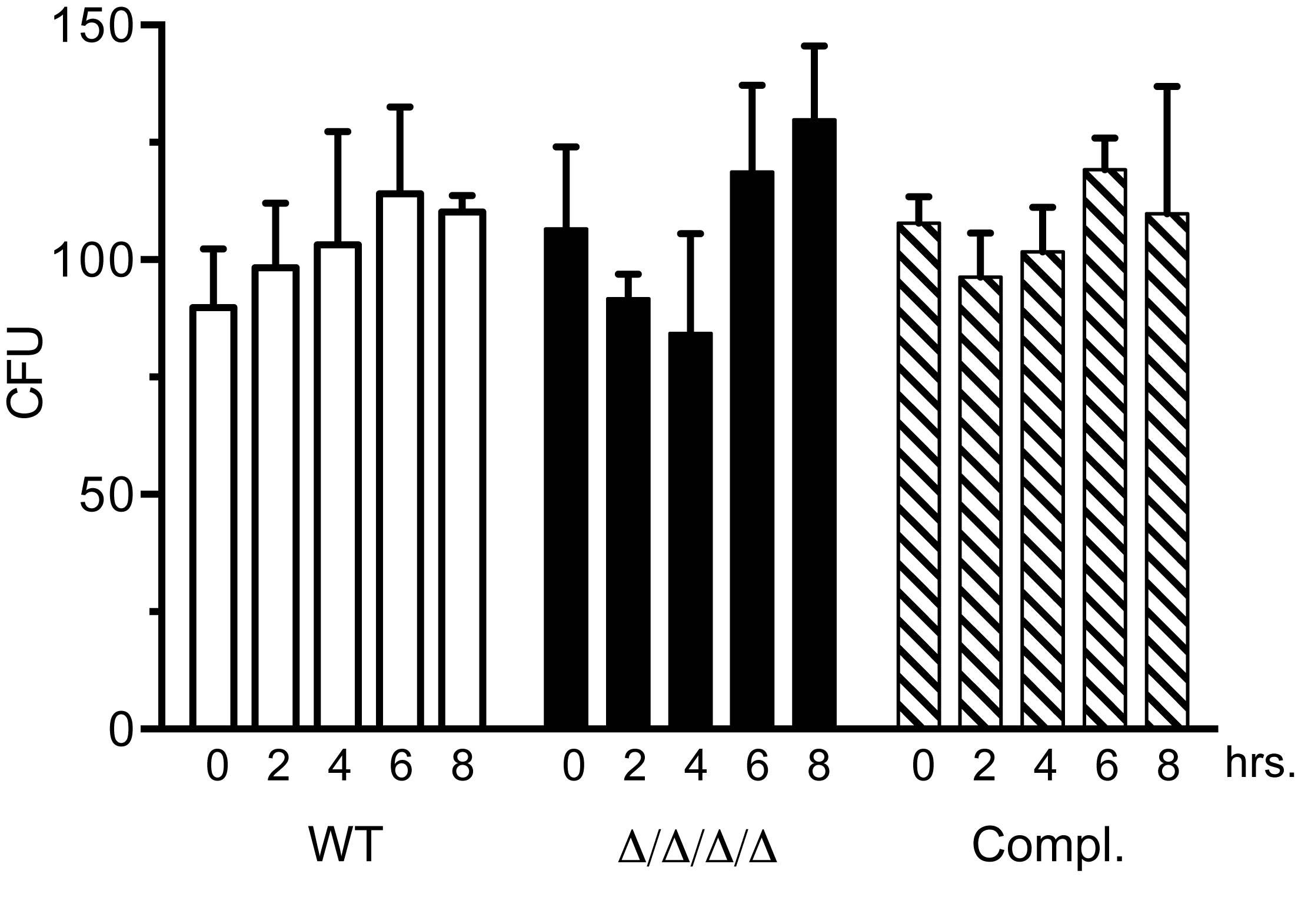

Supplement: S2 Fig — C. albicans strains were incubated with 0.5 mM oleic acid (OA) at 37°C for the indicated time (hours), and then dilutions of cells were plated to determine the viable colony forming units (CFU). These studies assays were carried out as controls for the cells incubated in the presence of linolenic acid (LNA) shown in Fig 2A. Strains used included the wild type strain LLF100, Δ/Δ/Δ/Δ strain LLF060, and the complemented strain LLF079 in which one copy of each FLP gene was introduced into the Δ/Δ/Δ/Δ strain. (TIF) [file ppat.1005147.s002.tif]

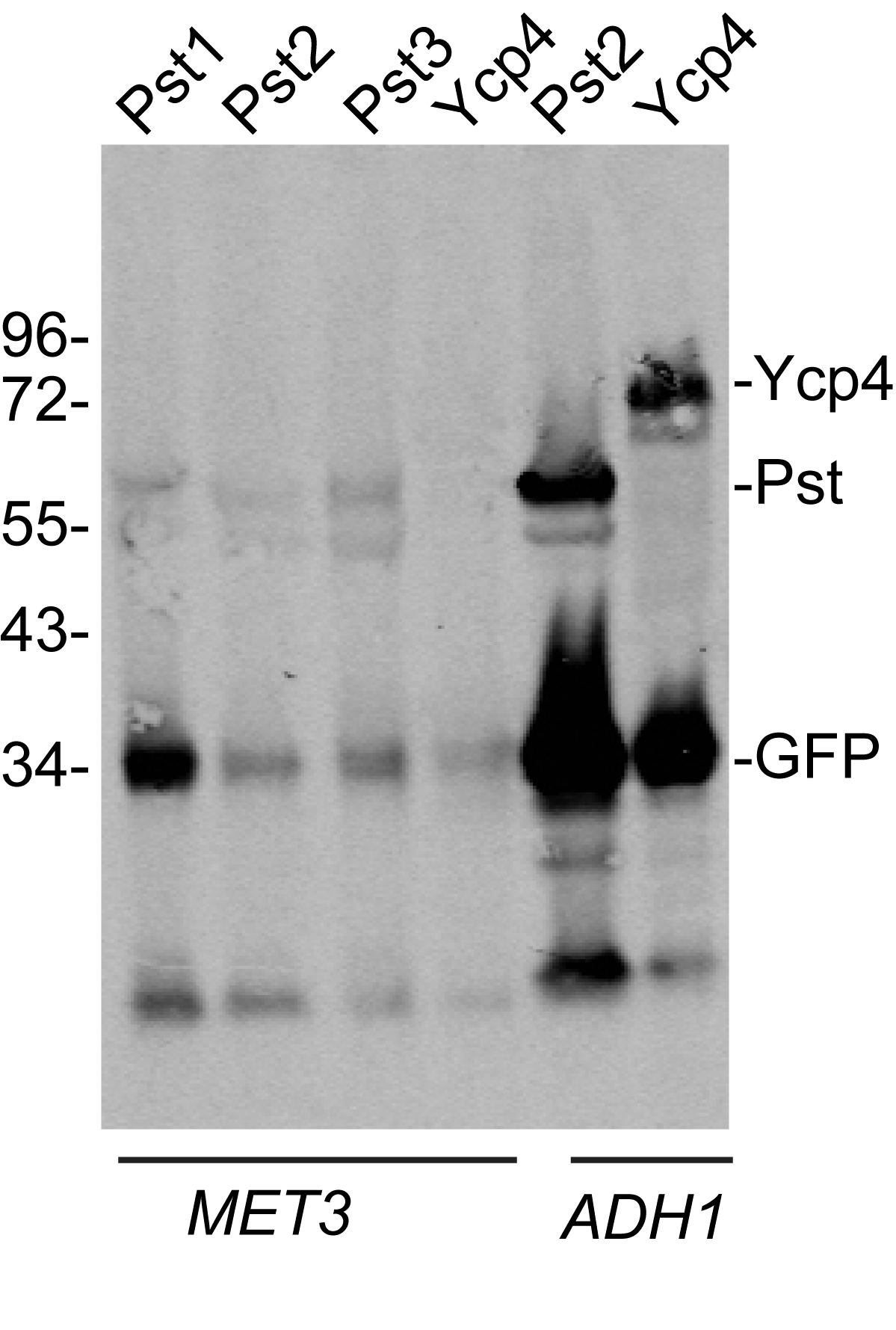

Supplement: S3 Fig — C. albicans cells containing fusions between GFP and the indicated FLP gene were analyzed on a Western blot probed with a mouse monoclonal anti-GFP antibody. The protein bands were detected using a secondary IRDye 800CW conjugated Goat (polyclonal) anti-mouse IgG antibody, and an image was acquired using a digital Odyssey infrared imaging system. To gain increased sensitivity, samples on the left side were expressed using a MET3 promoter and an ADH1 promoter was used for the samples on the right side, as indicated at the bottom. The position of pre-stained molecular weight markers (kD) is shown on the left side. The expected position for GFP is indicated on the right. Also on the right side, Pst indicates the approximate position for a GFP fusion to the Pst1, Pst2 or Pst3 proteins, and Ycp4 indicates the position for GFP-Ycp4. Note that Ycp4 is about 8 kD larger than the Pst1, Pst2, and Pst3 proteins and displays the expected difference in gel mobility on the blot. (TIF) [file ppat.1005147.s003.tif]

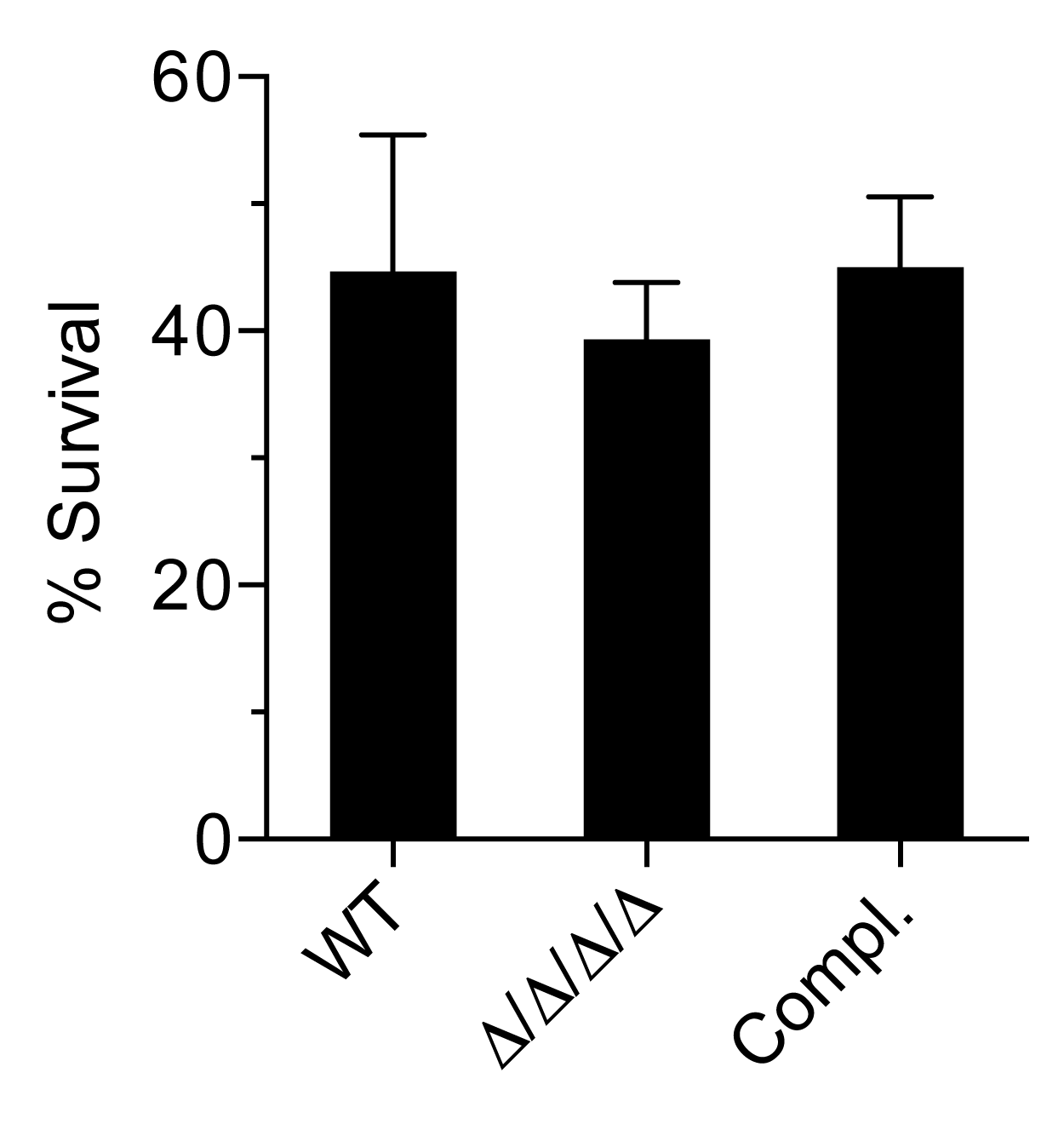

Supplement: S4 Fig — C. albicans strains were plated in multiwell trays in the absence or presence of macrophages derived from mouse bone marrow cells. Microcolonies of growth in each well were then counted to determine the reduction in C. albicans viability due to the presence of macrophages [73]. The results represent the average of three independent experiments. There was no significant difference in killing of the different C. albicans strains, as determined by ANOVA. Error bars indicate SD. (TIF) [file ppat.1005147.s004.tif]

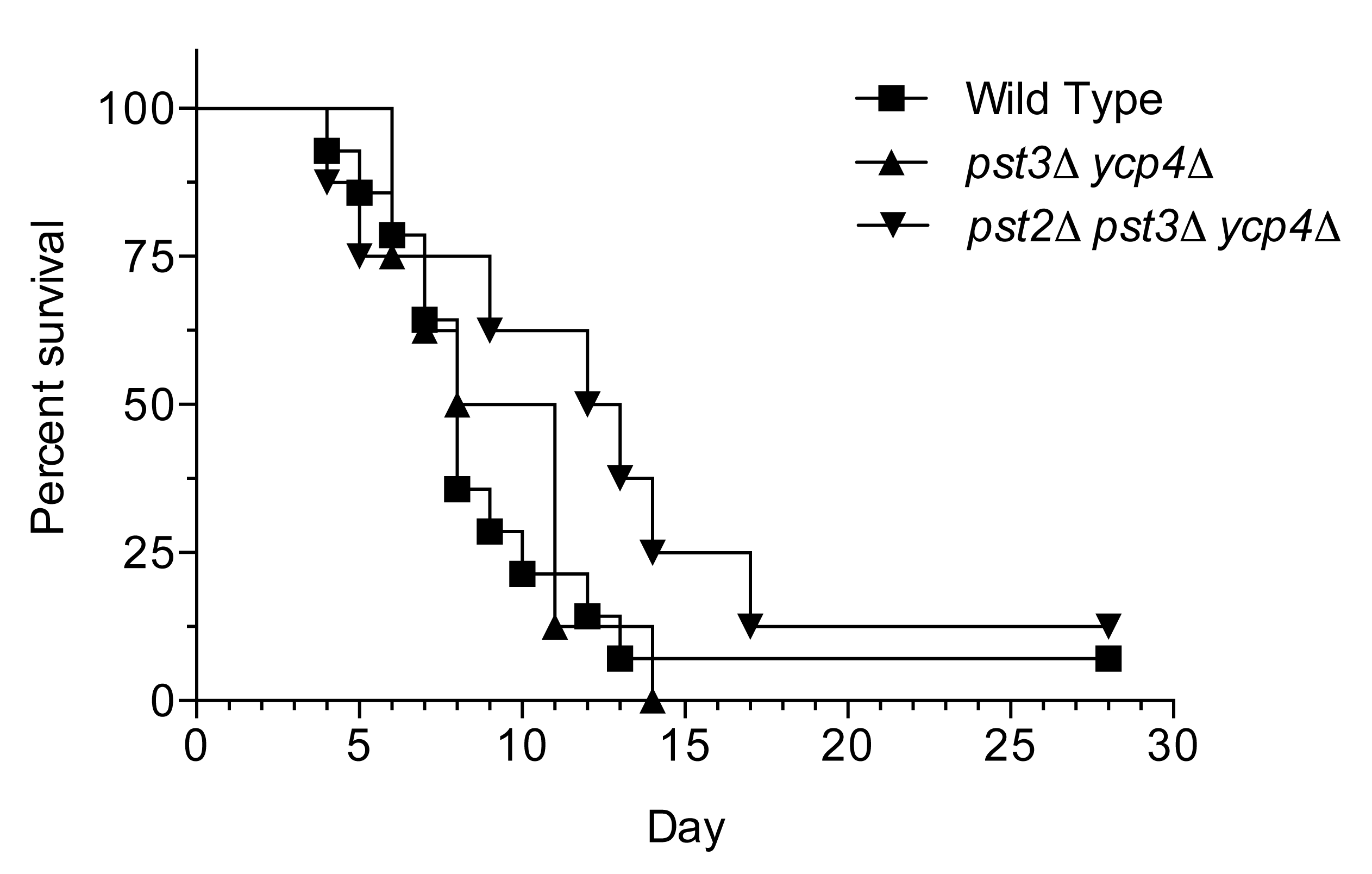

Supplement: S5 Fig — The double (pst3Δ ycp4Δ) and triple (pst2Δ pst3Δ ycp4Δ) FLP mutant C. albicans strains were assayed for virulence in a mouse model of systemic Candidiasis as described in Fig 8. Although mice infected with the triple mutant appeared to show slightly better survival, it was not statistically significant as judged by a log rank test (Mantel-Haenszel). Wild type control strain LLF100 was used to infect 14 mice, pst3Δ ycp4Δ strain LLF034 was used to infect 7 mice, and pst2Δ pst3Δ ycp4Δ strain LLF063 was used to infect 8 mice. (TIF) [file ppat.1005147.s005.tif]
